# Innate immune cell activation by adjuvant AS01 in human lymph node explants is age independent

**DOI:** 10.1172/JCI174144

**Published:** 2024-09-24

**Authors:** Vicki V. Stylianou, Kirstie M. Bertram, Van Anh Vo, Elizabeth B. Dunn, Heeva Baharlou, Darcii J. Terre, James Elhindi, Elisabeth Elder, James French, Farid Meybodi, Stéphane T. Temmerman, Arnaud M. Didierlaurent, Margherita Coccia, Kerrie J. Sandgren, Anthony L. Cunningham

**Affiliations:** 1 Centre for Virus Research, The Westmead Institute for Medical Research, Westmead, New South Wales, Australia.; 2 Sydney Medical School, Faculty of Medicine and Health, The University of Sydney, Sydney, New South Wales, Australia.; 3 Research and Education Network, Western Sydney Local Health District, Westmead, New South Wales, Australia.; 4 The Westmead Breast Cancer Institute, Westmead, New South Wales, Australia.; 5 GSK, Rixensart, Belgium.; 6 Center of Vaccinology, Department of Pathology and Immunology, Faculty of Medicine, University of Geneva, Geneva, Switzerland.

**Keywords:** Immunology, Vaccines, Innate immunity, Lymph, Macrophages

## Abstract

Vaccine adjuvants are thought to work by stimulating innate immunity in the draining lymph node (LN), although this has not been proven in humans. To bridge the data obtained in animals to humans, we have developed an in situ human LN explant model to investigate how adjuvants initiate immunity. Slices of explanted LNs were exposed to vaccine adjuvants and revealed responses that were not detectable in LN cell suspensions. We used this model to compare the liposome-based AS01 with its components, monophosphoryl lipid A (MPL) and QS-21, and TLR ligands. Liposomes were predominantly taken up by subcapsular sinus–lining macrophages, monocytes, and DCs. AS01 induced DC maturation and a strong proinflammatory cytokine response in intact LN slices but not in dissociated cell cultures, in contrast to R848. This suggests that the onset of the immune response to AS01 required a coordinated activation of LN cells in time and space. Consistent with the robust immune response observed in older adults with AS01-adjuvanted vaccines, the AS01 response in human LNs was independent of age, unlike the response to R848. This human LN explant model is a valuable tool for studying the mechanism of action of adjuvants in humans and for screening new formulations to streamline vaccine development.

## Abstract 2

Vaccine adjuvant AS01 initiates immune responses in adult human lymph nodes by activating innate immune cells and inducing proinflammatory cytokines, independent of donor age.

## Graphical abstract

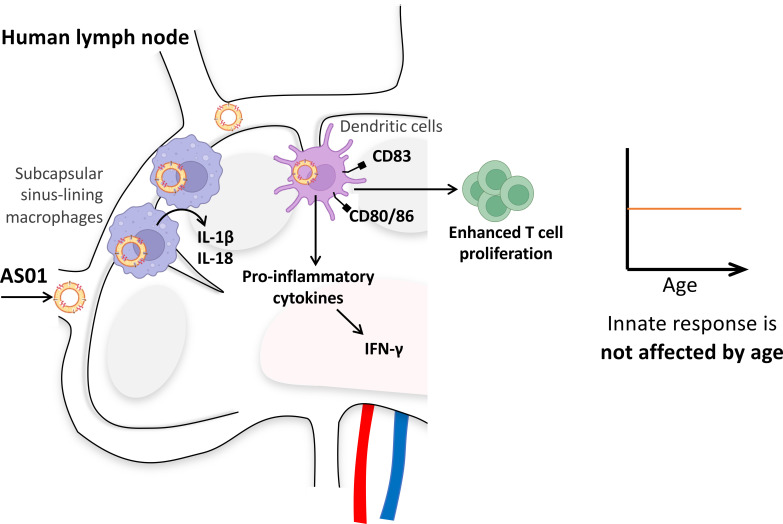

## Introduction

Incorporation of adjuvants into subunit vaccines has markedly increased the long-term immunogenicity and efficacy of these vaccines, particularly in aging and immune-compromised populations. An excellent example is the recombinant zoster vaccine (Shingrix, GSK), consisting of recombinant varicella zoster virus glycoprotein E (gE) and the adjuvant system AS01_B_, with high efficacy in all age groups, including those over 80 years of age (YOA), for at least 10 years (1–3). Phase I/II studies showed that the absence of AS01 reduced gE-specific CD4^+^ T cell responses by more than 10-fold in those over 70 YOA (4). AS01 consists of a TLR4 agonist, monophosphoryl lipid A (MPL), and the saponin QS-21 formulated in liposomes. The immunostimulants in AS01 act synergistically in mouse models to enhance CD4^+^ T cell responses (5). However, the recombinant zoster vaccine, as shown for other adjuvanted vaccines, results in an increased incidence of local and systemic symptoms occurring shortly after vaccination: 9%–11% of recipients experience reactions that prevent daily life activities, although these only last for 2–3 days (6). However, such reactogenicity is not correlated with immunogenicity (7), suggesting that adjuvants can be modified or developed to retain immunogenicity but with lower reactogenicity.

In order to achieve this, the exact mechanism of action in humans needs to be elucidated. For AS01, extensive studies have been conducted in mouse models, showing the rapid transit of AS01 and associated antigens to lymph nodes (LNs), where the onset of the immune response occurs (8). There the immune stimulants are taken up by sinus-lining macrophages, stimulating caspase-1 activation and IL-18 production. Early activation of macrophages initiates a cascade of immune responses including an early burst of IFN-γ from NK and CD8^+^ T cells in an IL-18– and IL-12–dependent manner. This culminates in DC activation and presentation of antigen to T and B cells, measured by a marked increase in antigen-specific antibody and polyfunctional CD4^+^ T cells (8–11). Some of these events have been confirmed in nonhuman primate LNs and human blood (11, 12). However, there are many differences between human and murine immune processes, including in LNs (13, 14), and findings in mice should be validated in humans.

Therefore, we have developed an in situ human LN explant model to study the mechanism of action of vaccine adjuvants, including AS01, for which abundant data exist in animal models. We found that liposomes of composition similar to that of AS01 were preferentially taken up by CD169^+^ sinus-lining macrophages and DCs. AS01 induced the maturation of DCs and the production of an array of proinflammatory cytokines including IL-1β, IL-18, and IFN-γ in intact LN slices, but not when LN cells were dissociated from tissue. DCs from AS01-exposed LNs also had an enhanced capacity for naive T cell stimulation. Unlike the LN response to other adjuvants, the response to AS01 was relatively independent of the adult LN donor’s age, which may underlie the remarkable efficacy of AS01-formulated vaccines in older adults (1, 15).

## Results

### Human LN explant model.

**Figure 1:**
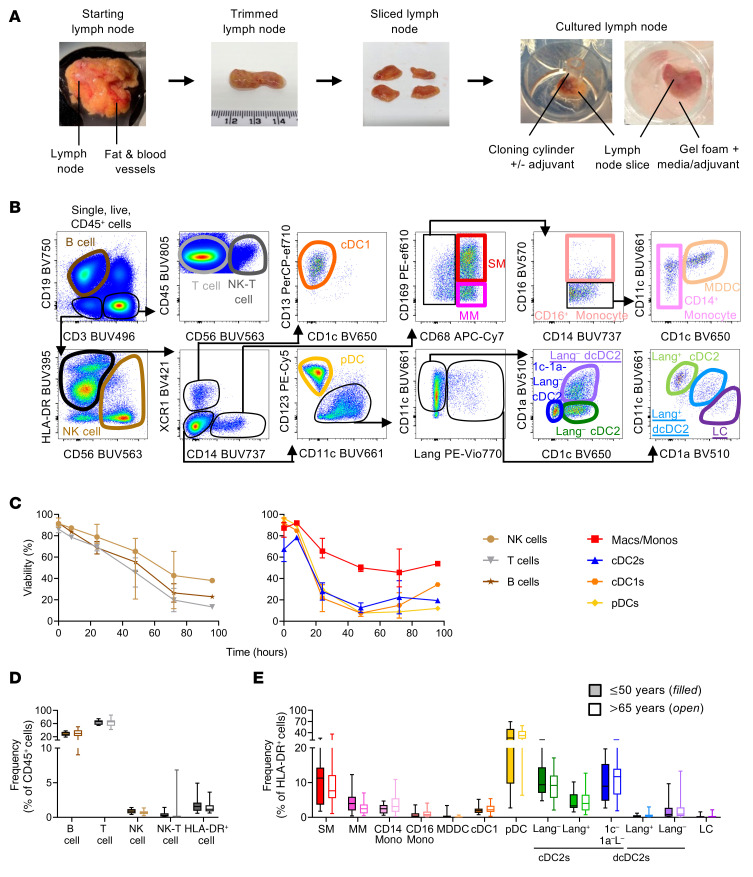
Human LN explant model for studying vaccine adjuvants in situ. (**A**) Fat is trimmed from human LNs, which are then sliced longitudinally and placed cut-face down on gel foam soaked in culture medium. For adjuvant exposure via the most physiological route (i.e., via the afferent lymph), a cloning cylinder is glued to the capsule of the LN and the adjuvant applied within this. For maximum cellular exposure, LN slices are bathed in the adjuvant. (**B**) Flow cytometric gating strategy used to identify LN cell populations, including resident and migratory (outlined) DC populations. (**C**) Viability of LN lymphocyte and myeloid cell populations following in situ culturing (*n* = 4 for 0-, 24-, 48-, and 72-hour time points; *n* = 1 for 8- and 96-hour time points) was measured by flow cytometry. The median with the IQR for available donors is shown for each cell subset at each time point. (**D**) Frequency of cell populations within fresh, uncultured LN with a comparison between donors aged 50 YOA or younger (filled boxes, *n* = 12) and older than 65 YOA (open boxes, *n* = 22). The median and IQR are shown for lymphocyte (B, T, NK, NK-T) and myeloid (HLA-DR^+^) cell populations as a percentage of live, CD45^+^ immune cells and (**E**) macrophage, monocyte, and DC subsets within the LN as a percentage of live, CD45^+^, CD19^–^, CD3^–^, CD56^–^, and HLA-DR^+^ myeloid cells. All subset comparisons between LNs from young or older individuals were not significant by Mann-Whitney *U* test using the Bonferroni-Dunn correction method for multiple comparisons. Macs, macrophages; Monos, monocytes; dcDC2, dermud-derived conventional DC type 2; Lang, langerin; LC, Langerhans cell.

To study the mechanisms of action of adjuvants in situ in human tissue, we developed a human LN explant model. Uninvaded human axillary LNs were obtained from female patients with breast cancer who were clinically node negative but undergoing sentinel node biopsies. Informed consent was obtained for the removal of an additional LN for this study. Donors between the ages of 30 and 96 years, with 53% being 60 YOA or older (Supplemental Figure 1; supplemental material available online with this article; https://doi.org/10.1172/JCI174144DS1). Longitudinal slices of whole human LNs, approximately 2 mm thick, were cultured on gel foam to provide hydration and structural support, which promoted cell viability (Figure 1A).

We used a high-parameter flow cytometry panel to detect all subsets of myeloid cells, which include resident DC subsets including conventional type 1 and type 2 DCs (cDC1, cDC2) and plasmacytoid DCs (pDCs), sinus-lining and medullary macrophages (SMs and MMs), and monocytes, as well as NK cells, NK-T cells, and B cells and T cells (Figure 1B). Migratory skin-derived DCs (CD11c^+^CD1a^+^langerin^+/–^) that were present in the LN at the time of excision were also detected. First, we assessed the viability of different cell populations in LN explant cultures by flow cytometry. T, B, and NK cells survived for 48–72 hours, but viability of the DCs and macrophages declined in the LN during the 24-hour culture period (Figure 1C). We detected fewer numbers of macrophages in particular after explant culturing, rather than appearing more strongly stained with the viability marker, indicating that they were lysing (Supplemental Figure 2A). Importantly, the viability of cells in LN slices cultured for 24 hours was similar regardless of whether the cultures were stimulated with adjuvant AS01. AS01 was well tolerated up to a concentration of 25 μg/mL in situ and in vitro, but higher concentrations decreased cell viability (Supplemental Figure 2). Furthermore, the viability of total dissociated LN cells, consisting of more than 98% lymphocytes, cultured in vitro for 24 hours (72.9% ± 17.0%, mean ± SD; Supplemental Figure 2C), was only slightly better than the viability of the lymphocytes cultured in LN slices (T cells 69.7% ± 7.6%, B cells 68.8% ± 11.8%; Figure 1C). As such, we limited explant cultures to 24 hours and included mock-treated controls to account for any effects produced by dying cells.

Next, we compared the immune cell constitution of LNs from young (50 YOA, *n* = 12) and older (>65 YOA, *n* = 22) individuals in fresh, uncultured LNs. LNs from young or older donors were remarkably similar. T and B cells represented the bulk of cells, with CD3^–^CD19^–^HLA^–^DR^+^ antigen-presenting cells (APCs) representing 1.79% ± 1.16% in LNs from young individuals and 1.43% ± 0.84% in those from older individuals (mean ± SD) (Figure 1D). The constitution of this HLA-DR^+^ population was also remarkably similar between the 2 age groups, with no significant differences in the cell subset proportions (Figure 1E).

### CD169^+^ sinus-lining macrophages preferentially take up AS01-like liposomes.

**Figure 2:**
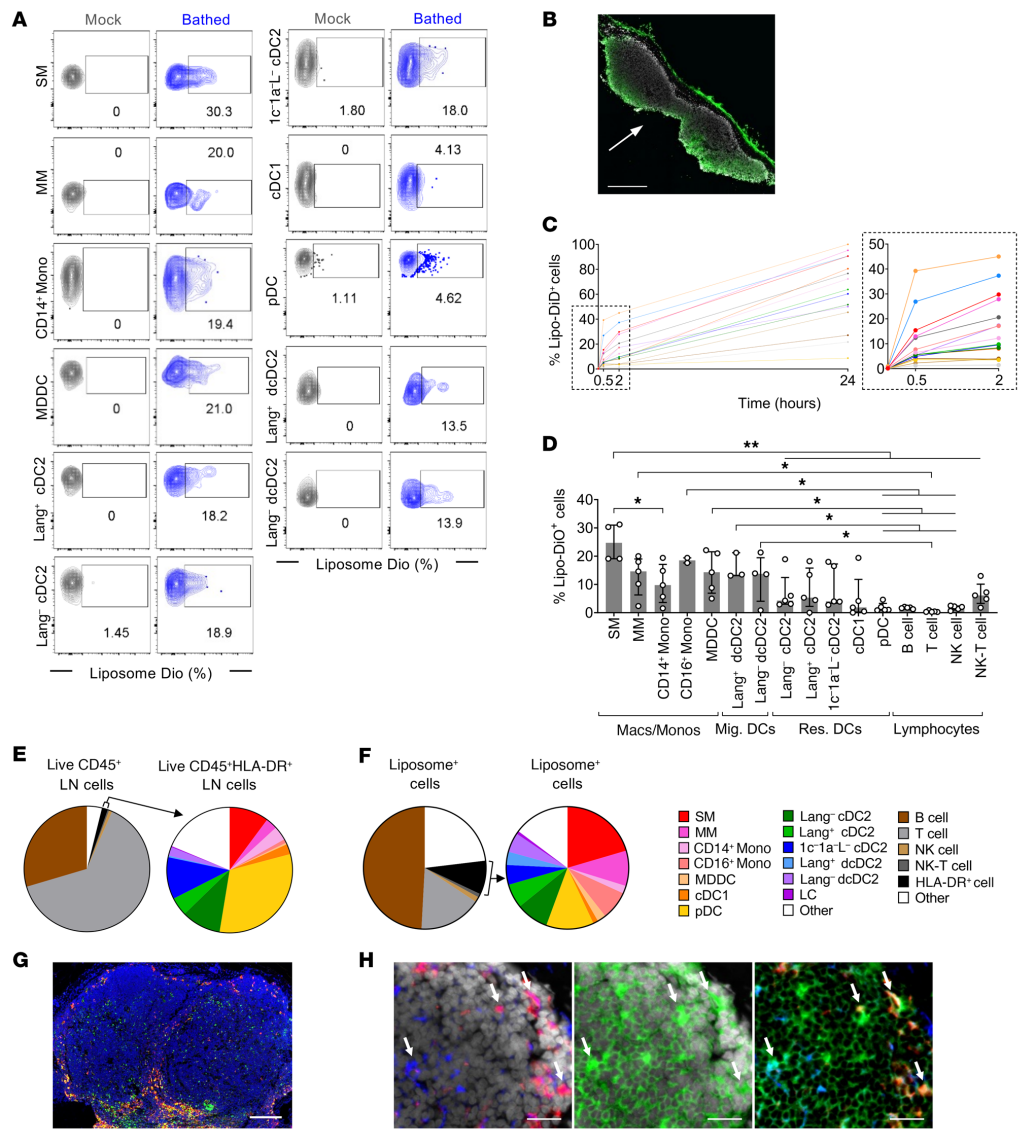
Fluorescent liposomes, a model for AS01, are preferentially taken up by subcapsular SMs in LNs. Slices of human LNs were exposed to DiO-, or DiD-labeled liposomes for 0.5, 2, or 24 hours. Cells were mechanically dissociated for flow cytometry, or the tissue was prepared for microscopy. (**A**) Representative flow cytometry plots showing DiO-liposome uptake after 2 hours of bathing, by resident myeloid cells and migratory skin-derived cells (*n* = 3–5). (**B**) Immunofluorescence image of LN slice showing DiD-labeled liposomes penetrated the tissue within 30 minutes of exposure. Arrow indicates exposed face. Scale bar: 500 μm. *n* = 3. (**C**) Uptake of DiD-labeled liposomes over 24 hours, measured by flow cytometry (*n* = 3). Colors in **C**, **E**, and **F** correspond to the cell subset legend. (**D**) Uptake at 2 hours was compared between LN cell subsets of the major groups: macrophages/monocytes, migratory DCs (Mig. DCs), resident DCs (Res. DCs), and lymphocytes. The median and IQR are plotted for each subset. Mixed-effects analysis with Tukey’s multiple-comparison test was performed. *P < 0.05 and **P < 0.01. For grouped statistical representation, the highest common *P* value is presented, but lower values were generated. (**E**) Immune cell subsets present in the LN (*n* = 50) and (**F**) making up the total liposome^+^ fraction after a 2-hour exposure (*n* = 5), showing cell subsets as a proportion of total live, CD45^+^ immune cells and myeloid cell subsets as a proportion of HLA-DR^+^ cells. (**G**) Mass cytometry image showing CD169 (red) and CD68 (green) staining in the LN. CD169^+^CD68^+^ SMs appear yellow; DAPI staining is shown (blue). Scale bar: 200 μm. (**H**) CD169^+^ (red) subcapsular SMs and CD11c^+^ (blue) DCs took up DiD-labeled liposomes (green) in situ in the LN after a 2-hour exposure (indicated by arrows). The capsule is visible at the top right. Scale bars: 25 μm. The median with the IQR for available donors is shown for each cell subset at each time point.

To assess the effects of adjuvant on LN explants, adjuvant treatments were applied via 2 routes (Figure 1A): a cloning cylinder glued to the capsule of the LN allowed the adjuvant to enter the LN probably via the afferent lymphatic vessels on the LN surface, as occurs in vivo, or possibly penetrating through the capsule directly. Alternatively, the cut face of the LN was exposed directly by placing it on gel foam soaked in adjuvant-containing culture medium (“bathing”; Figure 1A) and this allowed for maximum exposure of LN immune cells, which yielded stronger immune responses. To model the uptake of AS01 in situ in LNs, we used liposomes of equivalent composition and similar size, without MPL and QS-21 but incorporating the lipophilic fluorescent dye DiO or DiD. Slices of human LNs were exposed in situ to labeled liposomes via both the bathing and cylinder application methods for 30 minutes to 24 hours to determine the degree of liposome uptake for each immune cell subset by flow cytometry (Figure 2A). Immunofluorescence microscopy confirmed that liposomes penetrated the LN slice within 30 minutes via bathing (Figure 2B), and the degree of penetration increased over 24 hours (Supplemental Figure 3A). Correspondingly, uptake of liposomes by each cell type increased over 24 hours (Figure 2C). For all subsets, a larger percentage of cells were exposed to the liposomes via bathing compared with cylinder application, resulting in a higher degree of uptake, as shown by the percentage of cells that were liposome positive (Figure 2D, Supplemental Figure 3B). However, the distribution of liposome uptake across subsets was proportional (Figure 2, E and F, and Supplemental Figure 3C), with a strong correlation between the 2 exposure routes (Supplemental Figure 3D), indicating that the liposomes penetrated the LN effectively via both methods and that the bathed route did not introduce a bias on liposome uptake. We therefore conducted all experiments using the bathing exposure method as it (a) increased exposure of the cells to the liposomes and therefore presumably the adjuvant and (b) did not result in exposure of any cells that would not normally encounter the adjuvant when exposed via the physiological route simulated by the cloning cylinder.

The CD169^+^ SMs, found in both the medullary sinuses and the subcapsular sinus, had the highest capacity for liposome uptake at the single-cell level (Figure 2D), probably due to the superficial position of the subcapsular SMs lining the large peripheral sinuses of the LN (Figure 2G) and also their innate capacity for particle uptake. The accumulation of liposomes in the cytoplasm of CD169^+^ subcapsular SMs was confirmed by microscopy (Figure 2H). The remaining macrophage and monocyte subsets also had a relatively high capacity for liposome uptake, especially CD14^+^ monocyte-derived DCs (MDDCs). Of the DC populations, cDC2s were more efficient at liposome uptake than cDC1s, with migratory dermal cDC2s being better than resident cDC2s (Figure 2D). pDCs took up very little liposome. This hierarchy is consistent with the generally documented phagocytic capacity of these cells (16). These results are also consistent with reports in mice highlighting the role of subcapsular SMs in the initial uptake of AS01 and the critical role of subcapsular SMs and cDC2s in the initiation of the immune response (10, 11).

Liposomes were poorly taken up by lymphocytes, with only a modest increase in fluorescence over 24 hours (Figure 2C) that was likely only surface associated. Of the total liposome^+^ cells, HLA-DR^+^ APCs were overrepresented, at 8.93% ± 7.03% (mean ± SD, *n* = 5), despite representing only 1.55% ± 0.90% (mean ± SD, *n* = 49) of live CD45^+^ cells in the LN (Figure 2, E and F). Myeloid cells, particularly SMs, also preferentially took up liposomes compared with their proportion of the total cell population (Figure 2, E and F).

### AS01 induces maturation of DCs, but only in intact human LNs.

A key property of an adjuvant is the capacity to enhance the activation of APCs, inducing their upregulation of costimulatory molecules (CD80, CD83, CD86), which allow them to stimulate T cell proliferation. We assessed whether AS01, its components MPL and QS-21, or other TLR ligand adjuvants — R848 (TLR7/8) and Pam2Cys (TLR2) — induced maturation of LN myeloid cells and activation of NK cells and lymphocytes. Initially, to clarify the direct effect of AS01 on immune cells, cells were mechanically dissociated from LN tissue and stimulated as a mixed population with AS01 in vitro for 24 hours. MPL and R848 were included as comparators. AS01 induced no or very weak upregulation of the maturation markers CD80 and CD86 on dissociated DCs compared with donor-matched control samples. AS01 also did not activate lymphocytes or NK cells, measured by CD69 upregulation (Supplemental Figure 4). MPL was similarly nonstimulatory in vitro at a concentration equivalent to that of the MPL component of AS01. In contrast, R848 was potent at inducing upregulation of CD86 on macrophages, cDC2s (CD14^–^CD11c^+^ cells), and pDCs, and in 4 of 6 cDC1 donors, as well as upregulation of CD69 on NK cells, NK-T cells, and T cells and B cells (Supplemental Figure 4).

**Figure 3:**
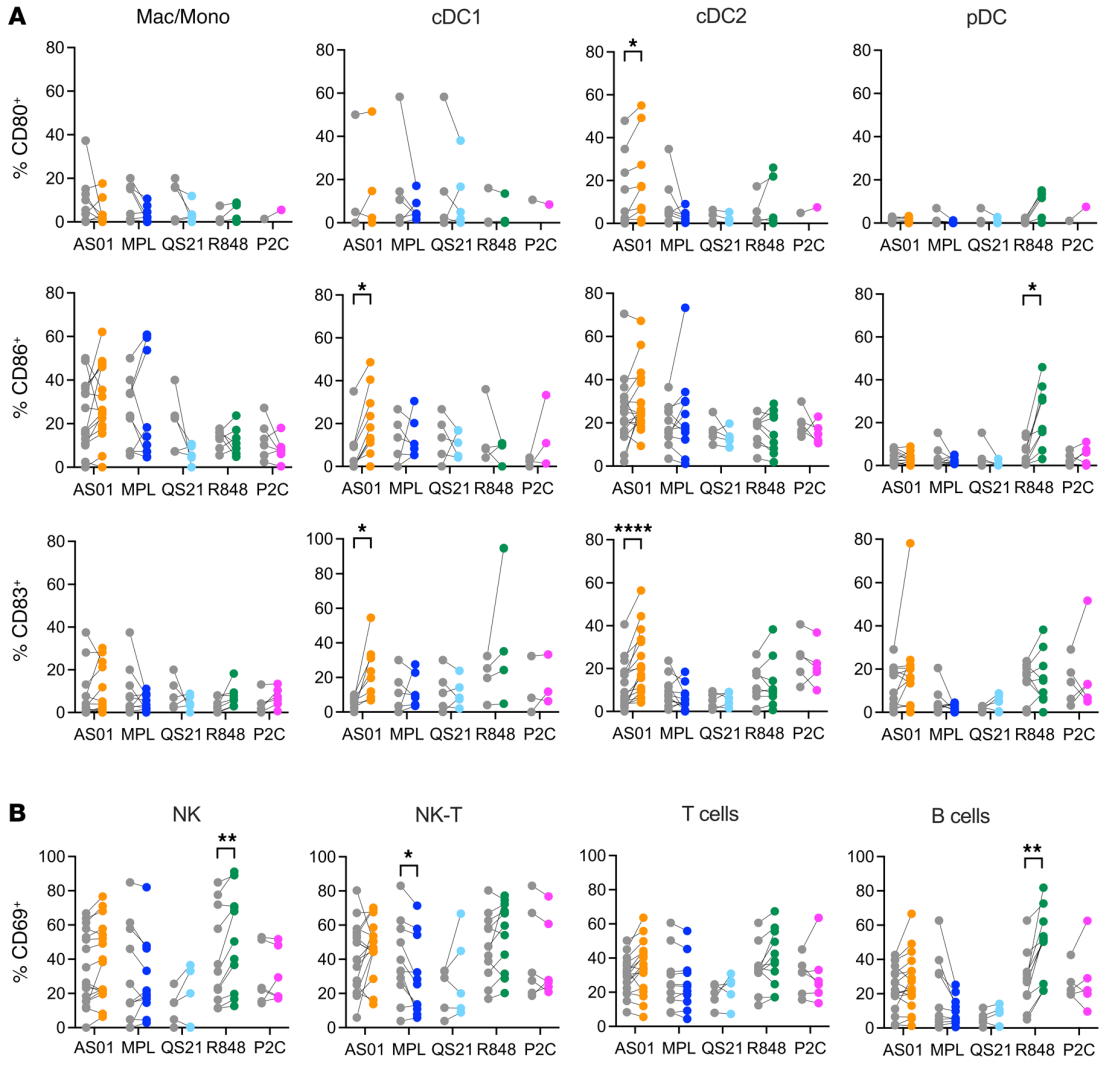
AS01 induces maturation of both cDC1s and cDC2s in situ in intact human LNs. Slices of human LNs were bathed in the adjuvants AS01 (orange), MPL (blue), QS-21 (light blue), R848 (green), or Pam2Cys (pink, P2C) or were mock treated (gray) for 24 hours. Cells were then mechanically dissociated from the LN tissue, and the percentage of (**A**) macrophage/monocyte and DC populations expressing the maturation markers CD80 (AS01, *n* = 3–10; MPL, *n* = 1–4; QS-21, *n* = 5; R848, *n* = 3–6; Pam2Cys, *n* = 1), CD83 (AS01, *n* = 8–17; MPL, *n* = 1–6; QS-21, *n* = 5; R848, *n* = 4–9; Pam2Cys, *n* = 3–6), and CD86 (AS01, *n* = 10–17; MPL, *n* = 1–6; QS-21, *n* = 5; R848, *n* = 4–9; Pam2Cys, *n* = 3–6), and (**B**) NK cells and lymphocytes expressing the early activation marker CD69 (AS01, *n* = 17; MPL, *n* = 6; QS-21, *n* = 5; R848, *n* = 11; Pam2Cys, *n* = 6) were assessed by flow cytometry. Wilcoxon matched-pairs, signed-rank tests were applied with Bonferroni-Dunn correction for multiple comparisons. *P < 0.05, **P < 0.01, and ****P < 0.0001.

In contrast to the in vitro results, AS01 did induce maturation of cDCs when intact LN slices were exposed in situ for 24 hours. We observed significant upregulation of CD83 and CD86 on cDC1s and CD80 and CD83 on cDC2s (Figure 3A). CD86 and CD83 were significantly upregulated when resident cDC2s were analyzed by subsets, including langerin^+^ and langerin^–^ subsets (Supplemental Figure 5). In some donors, rare DC subsets, such as cDC1s, could not be detected. Higher concentrations of AS01 tended to reduce cell viability (Supplemental Figure 2D) as well as the maturation response (Supplemental Figure 5B). Unlike AS01, the other stimuli — QS-21, MPL, R848, and Pam2Cys — did not consistently induce maturation of cDCs in situ, although R848 did mature pDCs, increasing their expression of CD86 (Figure 3A), and activated both NK and B cells (Figure 3B). The results of these 2 experiments show that AS01 induced the maturation of cDCs but only when the LN structure was intact. This suggests that, rather than directly activating DCs, AS01 induced this effect via an amplifying immune cascade that required not only the presence of multiple cell types but, critically, also the native structural organization of the LN. It also shows that lymphocytes were not directly activated by AS01. Conversely, R848 was more effective in directly activating TLR7/8-expressing cells in vitro than in the tissue, whereas QS-21, MPL, and Pam2Cys did not induce cellular maturation or activation by themselves.

### AS01 induces proinflammatory cytokines, but only in intact human LNs.

We assessed the cumulative production of proinflammatory cytokines in response to AS01, R848, and MPL, when dissociated LN cells were exposed in vitro, and in response to all 5 adjuvants when whole LN slices were exposed in situ for 24 hours. As with maturation, no proinflammatory cytokines could be consistently detected after AS01 stimulation of the total dissociated cell population in vitro (Supplemental Figure 6). IL-1β was detected in LN cells from 4 of 10 donors. Conversely, and consistent with its in vitro effect on maturation, R848 induced the inflammatory cytokines IFN-α, IL-1β, IL-18, IL-6, IL-8, and TNF, as well as antiinflammatory IL-10, and showed a trend for the induction of IFN-γ in LN cells from 5 of 8 donors. MPL was less inflammatory, significantly inducing IL-6 and IL-8 and inducing an increase in IL-1β and IL-18 in LN cells from 5 of 7 donors (Supplemental Figure 6).

**Figure 4:**
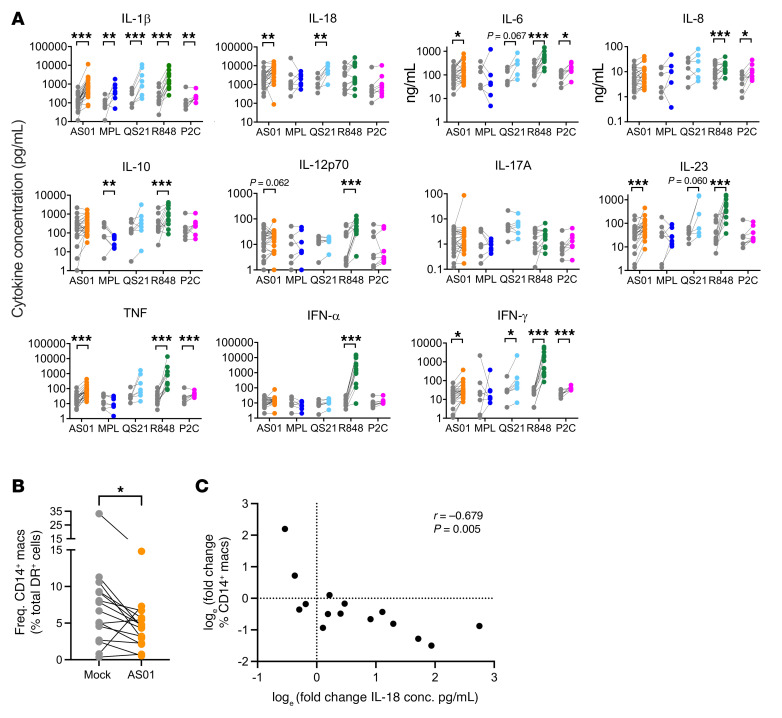
AS01 induces the production of proinflammatory cytokines in LN cells in situ. Slices of human LNs were bathed in adjuvants AS01 (orange, *n* = 27), MPL (blue, *n* = 8), QS-21 (light blue, *n* = 7), R848 (green, *n* = 13), or Pam2Cys (pink, *n* = 8) for 24 hours. (**A**) Cytokine concentrations in culture supernatants were determined by LEGENDplex and compared with their donor-matched, mock-treated (gray) samples. IL-6 and IL-8 were measured by ELISA. Data were log_e_ transformed to approximate normality, and GEE models were performed with a Bonferroni correction for multiple comparisons to compare donor-paired data. *P < 0.05, **P < 0.01, and ***P < 0.001. (**B**) Frequency (Freq.) of CD14^+^ cells in AS01 compared with mock-treated LN slices. *P < 0.05. (**C**) The fold change in CD14^+^ cell frequency negatively correlated with the production of IL-18. Pearson’s correlation was applied to the log_e_-transformed data. conc., concentration.

Although AS01 was rather inert in isolated cells, as with myeloid cell activation, we observed a much greater immune response in the more physiological in situ exposure model in terms of proinflammatory cytokine induction. We therefore focused on the in situ model and included QS-21 formulated in liposomes and Pam2Cys with the other stimuli. In situ, AS01 induced a range of proinflammatory cytokines*,* including IL-1β, IL-18, IL-6, IL-23, as well as TNF and IFN-γ (Figure 4A). We noted a trend toward increased IL-12p70 (*P* = 0.062), but IL-17A, IL-10, and IFN-α were not detected in response to AS01. Higher concentrations of AS01 did not increase the level of cytokine production (data not shown), consistent with the previously demonstrated decreased viability and maturation. MPL was again less inflammatory, only inducing IL-1β and downregulating IL-10. Cytokine induction by MPL was also compared with MPL formulated in liposomes and found to be comparable (Supplemental Figure 7). QS-21 in liposomes induced a cytokine profile similar to that of AS01, with the exception of TNF and IL-12p70. R848 induced an even broader range of cytokines in situ than in vitro*,* adding IL-12p70, IL-23, and clear induction of IFN-γ to its in vitro profile, although IL-18 was not significantly induced in situ. Pam2Cys was tested in situ, and although it did not induce cellular maturation or activation, it did induce a broad inflammatory response with IL-1β, IL-6, IL-8, TNF, and IFN-γ detected (Figure 4A). Furthermore, when a time course was performed, the induction of these cytokines was dynamic over 24 hours. For example, IL-1β and IL-18 were induced within 8 hours, and IFN-γ did not appear until 24 hours following AS01 stimulation (Supplemental Figure 8).

To summarize, in keeping with the maturation data, apart from IL-1β in some dissociated cell donors, AS01 only induced proinflammatory cytokines in intact LN tissue, again suggesting a requirement of the LN structure for the transmission of signals to multiple cell types upon exposure to AS01. QS-21 induced a proinflammatory cytokine response similar to that seen with AS01. At the concentrations tested, R848 was more immunostimulatory, activating several cell subsets directly, while MPL and Pam2Cys were less immunostimulatory, with moderate activation of the immune system both in vitro and in situ.

In mice, it has been shown that AS01 triggers an immune cascade, beginning with the activation of subcapsular SMs that produce IL-18. In synergy with IL-12, IL-18 rapidly enhances early IFN-γ production from NK and CD8^+^ T cells (10, 11). The production of IL-18 is linked to pyroptosis of the cell (17). In this study, after in situ AS01 exposure, the frequency of CD14^+^ cells, including macrophages, was significantly reduced (Figure 4B), and IL-18 production inversely correlated with the size of the CD14^+^ cell population (Pearson’s correlations *r* = –0.679, *P* = 0.005) (Figure 4C). Samples that had a strong upregulation of IL-18 in the supernatant had substantially depleted macrophage populations, with very few CD14^+^ cells and no discernible CD169^+^ SM population. The correlation of IL-18 production with the SM population was therefore weaker (*r* = –0.641, *P* = 0.010). Samples that had weak or no induction of IL-18 had much more robust populations, still smaller than the original population when the tissue was fresh, but distinct CD14^+^ and CD169^+^ cell populations remained. Therefore, it is likely that macrophages produced IL-18 in response to AS01 in situ, although they were dying in the process, as seen for QS-21 in mice (10). Increased IL-18 production, however, did not correlate with increased DC maturation (data not shown).

**Figure 5:**
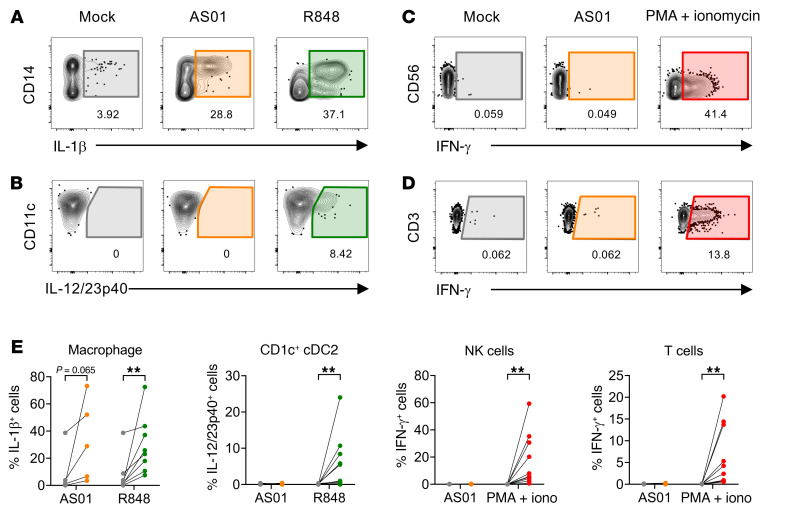
Macrophages produce IL-1β in response to AS01 in vitro, but downstream cytokines are not detected. Cells were dissociated from the LN and exposed in vitro to AS01, R848, or PMA/ionomycin for 24 hours in the presence of BFA. Production of IL-1β, IL-12/23p40, and IFN-γ was measured by flow cytometry. Representative data for (**A**) IL-1β and (**B**) IL-12/23p40 expression by total macrophages and CD1c^+^ cDC2s, respectively, as well as IFN-γ expression by (**C**) NK and (**D**) T cells, in response to mock or adjuvant treatments. (**E**) Percentage of macrophages expressing IL-1β, DCs expressing IL-12/23p40, and NK and T cells expressing IFN-γ in response to AS01 (IL-1β, *n* = 5; IL–12/23p40, *n* = 10; IFN-γ, *n* = 13), R848 (IL-1β, *n* = 8; IL-12/23p40, *n* = 12), and PMA plus ionomycin (Iono) (IFN-γ, *n* = 10). Wilcoxon matched-pairs, signed-rank tests were applied. **P < 0.01.

To confirm the discrepancy between our in vitro and in situ results, we directly assessed a range of cytokines by ICS of dissociated human LN cells in vitro. IL-18 production is difficult to detect by ICS due to the induction of pyroptosis, as mentioned above. IL-1β, clearly induced in situ by AS01 in human LNs, although only detected in very low amounts in mice upon AS01 administration (8), is produced by a common activation pathway to IL-18, but we could detect this by ICS. R848, included as a comparator, induced IL-1β production by CD14^+^ cells, which included macrophages, and IL-12/23p40 production by CD1c^+^ cDC2s. NK and T cells could produce IFN-γ in response to PMA/ionomycin stimulation. In contrast, AS01 induced IL-1β in macrophages from 5 of 5 donors but did not induce IL-12/23p40 in cDCs or IFN-γ in NK or T cells from any donors (Figure 5, A–E). These results were the same regardless of whether brefeldin A (BFA) was added early (2 hours into the culture, potentially blocking the early release of cytokines and their downstream effects such as IFN-γ induction) or late (8–12 hours into the culture, allowing more time for the full cytokine cascade before its addition), and therefore the combined data are shown (Figure 5E).

The induction of IL-1β and IL-18 in situ and at least IL-1β in vitro is consistent with the early AS01/QS-21 cytokine cascade shown in mice (8, 10, 11, 18), however, in humans when cells are dissociated from the LN, the downstream parts of the AS01 cytokine cascade are lost, highlighting an important role for this LN explant system in preserving the cell-cell contact required for the native immune responses.

### Age does not influence basal levels of DC maturation or cytokine release in the LN but does influence the DC response to adjuvants.

Aging results in disturbances in the structure of LNs, disorganization of the internal zones, and impaired intercellular interactions and cytokine responses (19, 20). Together with a reduced thymic output of naive T cells, these changes result in impaired immune responses to pathogens and vaccines.

**Figure 6:**
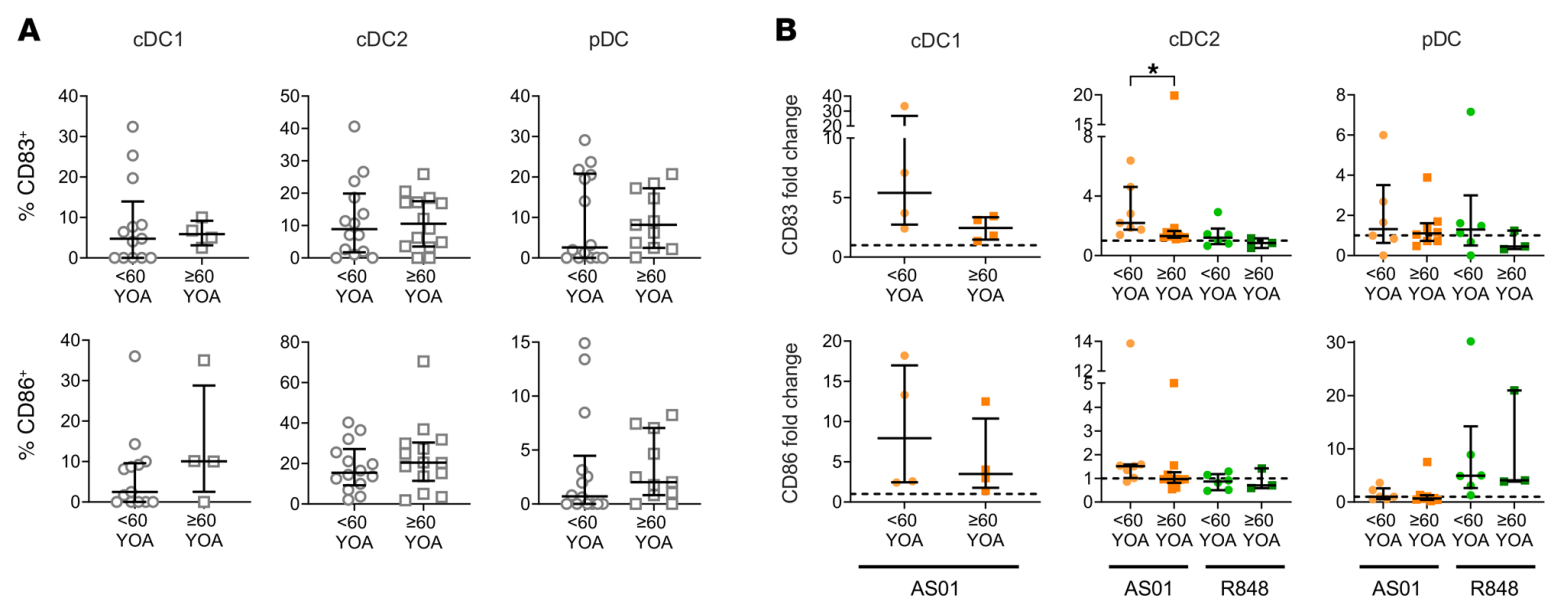
Expression of costimulatory molecules on DCs at baseline and in response to AS01 and R848 was similar between younger and older LN donors. Slices of human LNs were processed immediately (gray) and/or stimulated in situ for 24 hours with AS01 (orange) or R848 (green). Cells were mechanically dissociated from the LN tissue, and expression of CD83 and CD86 was assessed by flow cytometry. Comparisons were made between young (<60 YOA, circles) and older (≥60 YOA, squares) donors. (**A**) Percentage of CD83 and CD86 expression (LNs from young donors, *n* = 12–14; LNs from older donors, *n* = 4–14) at baseline. (**B**) Fold change in expression of CD83 and CD86 in response to AS01 (LNs from young donors, *n* = 4–7; LNs from older donors, *n* = 4–10) or R848 (LNs from young donors, *n* = 3–6; LNs from older donors, *n* = 0–3) compared with donor-matched mock samples. Not all of these samples were measured at baseline and vice versa. Medians with IQRs are indicated throughout. The median with the IQR for available donors is shown for each cell subset at each time point Mann-Whitney *U* tests were applied for each treatment. *P < 0.05.

In terms of functional effects, we did not observe a difference in the basal expression of the costimulatory molecules CD83 or CD86 on cDC1s, cDC2s, or pDCs from older (≥60 YOA) compared with younger adults (<60 YOA) (Figure 6A). Furthermore, cDC2s from younger donors upregulated CD83 more than did those from older donors in response to AS01, but otherwise there was no difference in the capacity of DCs to mature in response to AS01 or R848 in situ (Figure 6B).

**Figure 7:**
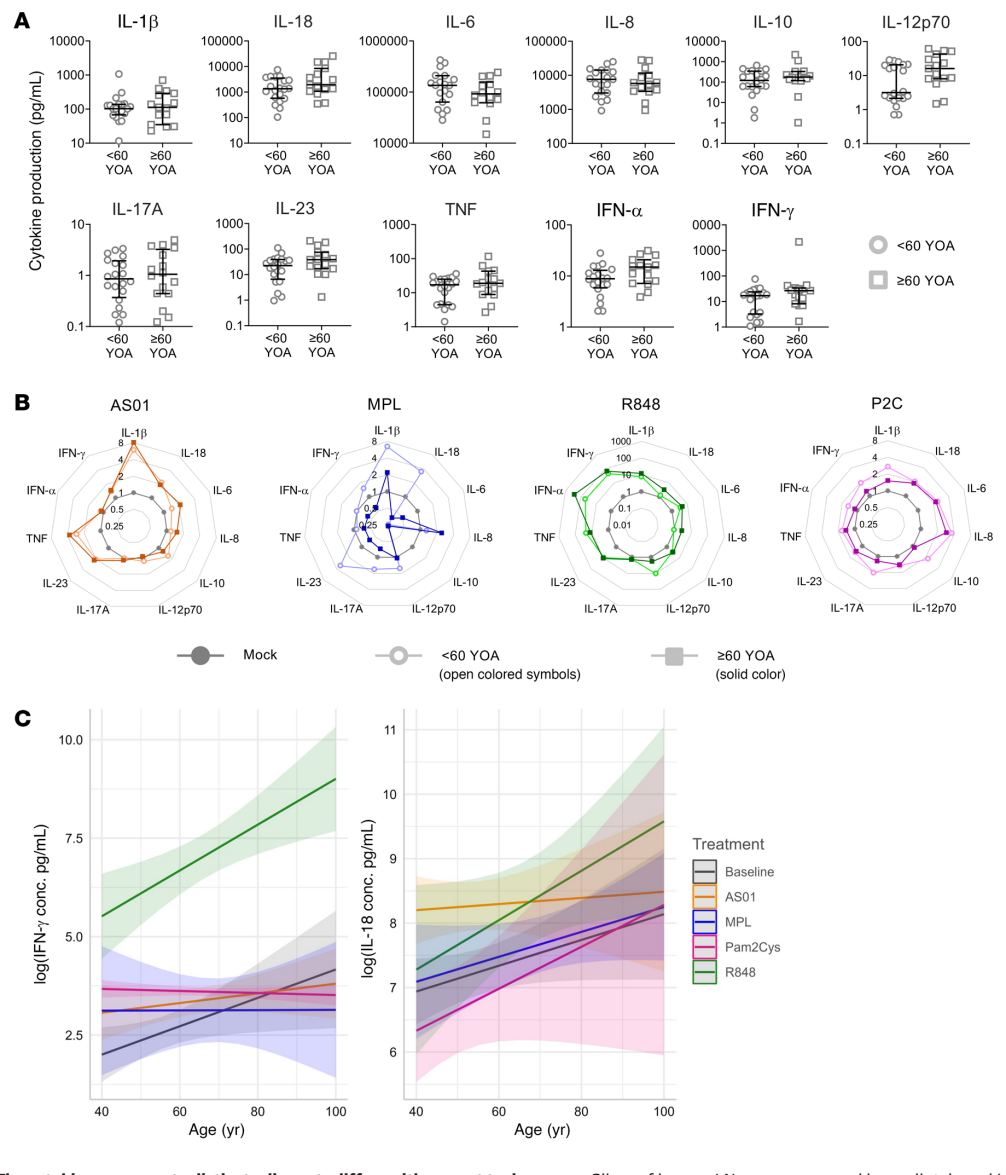
The cytokine response to distinct adjuvants differs with respect to donor age. Slices of human LNs were processed immediately and/or stimulated in situ for 24 hours with AS01 (orange), MPL (blue), R848 (green), or Pam2Cys (P2C) (purple) or left unstimulated (gray), and comparisons were made between samples from young (<60 YOA, circles) and older (≥60 YOA, squares) donors. (**A**) Level of cytokines in unstimulated cultures (LNs from young donors, *n* = 19–20; LNs from older donors, *n* = 14–15). Medians with the IQR are indicated. (**B**) Plots showing the median fold change in cytokine production in response to adjuvants (colored) compared with donor-matched mock samples (gray): AS01 (<60 *n* = 11; ≥60 *n* = 12–13), MPL (<60 *n* = 5; ≥60 *n* = 1–2), R848 (<60 *n* = 6–7; ≥60 *n* = 4), and Pam2Cys (<60 *n* = 5; ≥60 *n* = 3). Mann-Whitney *U* tests corrected for multiple comparisons using the Bonferroni-Dunn method were applied. (**C**) IFN-γ and IL-18 had a significant interaction with age in a GEE model (*P* ≤ 0.01). The amount of cytokine produced in response to each adjuvant is plotted with respect to the age of the LN donor: baseline (*n* = 34–36), AS01 (*n* = 24–25), MPL (*n* = 6–7), R848 (*n* = 12), and Pam2Cys (*n* = 8). The median with the IQR for available donors is shown for each cell subset at each time point.

Whereas increased circulating levels of TNF, IL-1β, and IL-6 have been reported in aging adults above 65 YOA compared with young adults under 30 YOA (21), we did not observe this in our cohort in unstimulated LN slices. Initially, in a univariate analysis, we found no difference in the basal levels of these or other proinflammatory cytokines in the supernatants of cultured LNs from older or younger donors (Figure 7A). As with maturation, we also did not find a significant difference in the capacity of older LNs to respond with proinflammatory cytokines to any of the adjuvants when comparing the median fold change (Figure 7B and Supplemental Figure 9). To further investigate the effect of aging, we used a robust general estimating equation (GEE) model to cluster readings by donor, considering age as a continuous variable. Here, we identified an interaction between age and adjuvant for IFN-γ and IL-18 (Figure 7C and Table 1), indicating that adjuvants had different effects on cytokine production depending on the age of the individuals. A natural increase in IFN-γ production and IL-18 production was observed with age in mock-stimulated cultures, consistent with inflammaging (22) and previous reports (23, 24), e.g., each YOA confers an additional immune response of 0.027 log IFN-γ units (Table 1). IFN-γ production in response to R848 strongly and significantly increased with age, although the opposite has been reported in blood (21). IFN-γ production in response to AS01 only increased slightly and was not significantly different from the natural increase observed with age alone. These 2 adjuvants differed from MPL and Pam2Cys, in which there was no age relationship for the IFN-γ response. The IL-18 response to R848 and Pam2Cys increased with age at a similar rate, but only the R848 response was significantly greater than the natural increase. The response to MPL also only increased in line with the natural increase. In contrast, the age-related increase in IL-18 in response to AS01 was slower than the natural increase with age, although their CIs slightly overlapped (Table 1). Thus, we observed differential responses to TLR ligands with age, but a consistent AS01-induced proinflammatory response was maintained in LNs from younger and older adults.

### AS01 enhances the capacity of DCs to stimulate naive CD4^+^ T cells.

**Figure 8:**
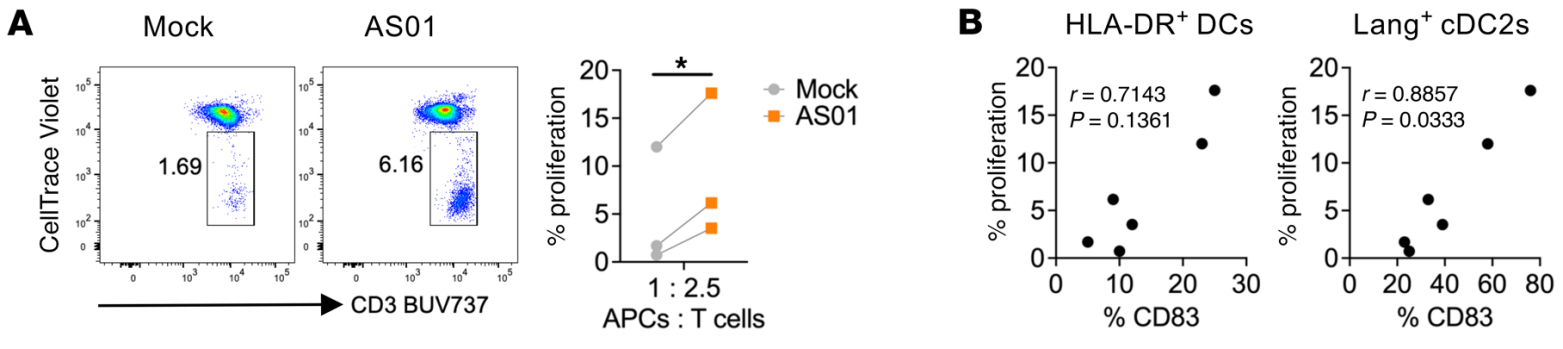
Proliferation of naive CD4+ T cells induced by AS01-exposed DCs. DCs isolated from slices of human LNs that were stimulated in situ for 24 hours with AS01 or mock conditions were cocultured with CellTrace Violet–labeled heterologous naive CD4^+^ T cells at a ratio of 1:2.5 for 5 days. (**A**) T cell proliferation was measured by CellTrace Violet dilution. *P < 0.05, by Student’s *t* test. (**B**) Correlation analysis of T cell proliferation with CD83 expression on total sorted DCs and subsets from mock- and AS01-treated samples was done with Spearman’s test. *n* = 3 biological replicates.

To explore the functional implications of the innate immune activation induced by AS01, we assessed DCs primed in AS01-exposed LN slices for their capacity to induce proliferation of heterologous naive CD4^+^ T cells. The latter have a higher threshold for activation than memory T cells, and their stimulation is important for both initial and booster vaccine doses (25, 26). AS01 primed DCs with enhanced antigen presentation capacity compared with mock-stimulated LNs (Figure 8A). The degree of proliferation correlated with the AS01-induced maturation (CD83 expression) of a subset of DCs, langerin^+^ cDC2s, with a trend toward a correlation with maturation of total DCs (Figure 8B and Supplemental Figure 10).

## Discussion

Predictive preclinical models to define the immunogenicity and mechanisms of action of vaccines and adjuvants in humans could be instrumental in the iterative development of new vaccines. Here, we have demonstrated the utility of a human LN explant model for investigating in situ innate immune responses to vaccine adjuvants. In this model, whole tissue slices were used to preserve the complex internal structure of the LN, including the capsule, at a thickness designed to maximize the representation of all compartments and the number of rare APC subsets that may be lost in small tissue blocks or thin slices. Cascading immune responses were preserved that were otherwise lost in dissociated cells, demonstrating the physiological relevance of the model and the importance of maintaining the spatial organization of cells and extracellular matrix structures within the organ. This model can be used as an additional tool for in vivo mouse and nonhuman primate models to test the mechanisms of action of existing and novel vaccines and adjuvants and their immunostimulatory properties at the very site where they work in vivo after intramuscular injection. With this human model, we investigated the innate immune response to AS01 spanning the initiating events through to the interface of innate and adaptive immunity. We describe the key LN cells that were targeted and stimulated by AS01 and demonstrate the functional consequence of this. Liposomes with a composition similar to that found in AS01 were preferentially taken up by subcapsular SMs and DCs, with SMs likely being the initial cells to respond. AS01 induced the maturation of multiple subsets of DCs, as well as the production of proinflammatory cytokines from multiple cell types in in situ–exposed LN slices but not in dissociated LN cell cultures. This led to DCs with enhanced potency for stimulating naive CD4^+^ T cell proliferation. The age of the adult LN donor did not influence the production of cytokines in response to AS01, unlike other adjuvants. This may be one factor underlying the efficacy of AS01-formulated vaccines, e.g., for herpes zoster and respiratory syncytial virus in older adults (1, 15).

We made several findings that demonstrated strong similarities between the mode of action of AS01 in mice and humans: (a) pattern of uptake in LNs, primarily by CD169^+^ subcapsular SMs but also DCs; (b) activation of APCs — macrophages and DCs; (c) initiation of a cytokine cascade that culminates in the early production of IFN-γ. The latter is likely produced by NK or CD8^+^ T cells.

Particles of approximately 10–100 nm in size can flow freely to the LN via the lymphatics (27), and AS01 is approximately 100 nm in size (28). Indeed, in mice, QS-21 in liposomes drains to the LN via the afferent lymphatics within 30 minutes of administration and is taken up by CD169^+^ macrophages that line the sinuses of the LN, including the subcapsular sinus, that are ideally positioned to sample lymph-borne antigen (10). These subcapsular SMs play an important role in transferring captured antigen to, and activating, B cells and produce an array of cytokines to coordinate multiple LN-resident immune cells (29). The uptake of our empty liposomes by subcapsular SMs and also DCs is consistent with this, and the pattern was the same whether liposomes were applied by a cloning cylinder to the external surface, or by bathing the entire cut surface of the explant. The latter may be explained by the size of the liposomes. Particles larger than 10 nm are too large to flow through the narrow lymphatic conduits to access the paracortex with its T cells and DCs (27), however, they can access the subcapsular SMs, DCs, and other cells in the superficial interfollicular cortex via the wider peripheral sinus and limited percolation into the tissue. AS01, being slightly smaller than our empty liposomes, may penetrate deeper into the cortex and paracortex. Thus, the superior liposome uptake by CD169^+^ subcapsular SMs is probably due to a combination of their advantageous location and inherent endocytic capability.

The adjuvanticity of AS01 in mice is in part due to the activation of DCs (8, 30). AS01 activated macrophages and cDCs in situ in the human LN model, inducing upregulation of costimulatory molecules. R848, a TLR7/8 ligand, did not mature cDC2s in situ, even though they express TLR8 and they were activated in vitro. R848 did mature pDCs, which express high amounts of TLR7, as well as NK and B cells (both TLR7^+^). R848, a small-molecule immune potentiator, would be able to penetrate the LN thoroughly but may have a stronger affinity for TLR7 than TLR8, or the lack of cDC2 activation may be a dose effect, with R848 being diluted in the LN explants.

A key cytokine axis in the AS01 response in mice is the IL-18– and IL-12–dependent induction of IFN-γ (11). QS-21 has been shown to activate the NLRP3 inflammasome, resulting in IL-1β and IL-18 production (10, 18), although in response to AS01, IL-1β has only been detected at very low levels in mice (8), and whether AS01 activates the inflammasome is still unclear. Our findings in human LNs that AS01 induced IL-1β, IL-18, IFN-γ, and in somedonors, also IL-12, with different kinetics over 24 hours, supports the idea of a similar cytokine cascade in humans that likely begins with inflammasome activation in macrophages and culminates in the production of IFN-γ. Interestingly, QS-21 alone did not induce TNF or IL-12, and the induction of these 2 cytokines may be a key feature of the interaction between MPL and QS-21 in AS01.

As we found, stronger and broader cytokine responses in intact lymphoid tissue slices compared with dissociated cell cultures have also been observed before (31–33). The bioavailability, and therefore potency, of an adjuvant used at similar doses should be higher in dissociated cultures than in in situ cultures, so the phenomenon likely relates to the need for complex cell-cell interactions along the reticulin framework (34, 35) as well as cytokine signaling. 3D cytokine gradients will be established much more effectively when the producing cells are fixed in place or by utilizing the LN architecture to move in a deliberate direction, as in intact tissue, rather than floating freely. IFN-γ–producing NK and CD8^+^ T cells may also need to be in close proximity to IL-12 and IL-18 production, which suggests a compartmentalization of the AS01 response to the subcapsular region of the LN.

Aging and immunosenescence have a detrimental effect on vaccine responses, with the efficacy of most vaccines being reduced in people over 65 YOA (36–39). However, there were vast differences between the recombinant zoster vaccine and the live herpes zoster vaccine, with efficacy decreasing markedly with age for the latter vaccine, especially over time (40). The AS01-adjuvanted recombinant zoster vaccine and the respiratory syncytial virus vaccine have overcome this (1–3, 15), but why this adjuvant is so effective in immunogenicity and efficacy in older adults is unknown (4, 41). We found that the immune constitution of old and young LNs was remarkably similar even with a 15-year age gap buffer. This is consistent with reports that the number and phenotype of circulating DCs are comparable in healthy older adults (42, 43) and young adults, apart from frail older individuals (44). This similarity implies that any differences in adjuvant immune responses observed between young and old donors would likely be due to differences in the functional capacity or interactions of immune cell subsets or the structure of the LN between young and older people, rather than differences in the frequency of individual cell populations. In the clinical trials of recombinant zoster vaccine, there were no sex-specific effects identified at any age, including in individuals above 70 YOA (45). Therefore, even though only LNs from female donors were tested in this study, in this setting the effect of age is clearly more important than sex.

Although inflammaging, the age-related increase in inflammation, is characterized by an increase in the circulating levels of the proinflammatory cytokines IL-1β, IL-6, and TNF and reduced levels of antiinflammatory cytokines such as IL-10 (46, 47), we did not observe these changes in the LNs from donors older or younger than 60 YOA. The increase in circulating proinflammatory cytokines in older adults may be driven by altered gut integrity or microbiota, or adipocytes, which increase with age and are another source of proinflammatory cytokines (48, 49). Our GEE model for testing the effect of age on adjuvant-induced cytokine production was influenced by a paucity of donors at the far ends of the age spectrum as well as the expected wide human donor variability of cytokine production, and thus had wide CIs. Nonetheless, IL-18 and IFN-γ pathways were found to be particularly conserved across the adult age spectrum compared with other adjuvants, which may contribute to the efficacy of AS01 in older adults. This is consistent with a recent report that found no age-related differences in response to AS01 in human blood myeloid cells (50), although as PBMCs are phenotypically and functionally divergent from LN cells (51), it is important to study both.

Using this in situ culture method for LN slices, we have demonstrated a dynamic innate immune response to AS01 over 24 hours, with cytokines being produced with different kinetics and the activation of DCs with the functional capacity to stimulate naive CD4^+^ T cells. T cell proliferation was most closely correlated with maturation of the langerin^+^ cDC2 subset. Notably, cDC2s have been identified in mice as necessary for the induction of adaptive immunity by AS01-containing vaccines, and AS01 is associated with potent activation of these cells (30). Also langerin^+^ cDC2s have a higher intrinsic level of *ICAM1* (CD54) expression than do langerin^–^ cDC2s in anogenital mucosa, which is critical for DC–T cell interactions, and are the most efficient at transferring HIV to CD4^+^ T cells (52), which suggests their particular efficiency in interacting with T cells.

Our model has several limitations. The viability of thick tissue explants is difficult to maintain ex vivo, with deeper parts of the tissue affected by hypoxia and diminished nutrient supply. The duration of viability of myeloid and lymphoid cells after isolation from our cultured 2 mm thick LN slices varied but was consistent with findings in similar models (33, 53, 54) and sufficient to allow determination of their early function in response to various adjuvants. Isolation of the cells from tissue after culture is stressful on the cells, and it may be possible to observe and measure immune responses in situ for longer than 24 hours. Smaller tissue blocks have been reported to remain viable for 3 weeks or more (55) but will often not contain the full gamut of sparsely distributed innate and stromal cells and be mostly composed of lymphocytes. Our model will require modification, such as judicious cytokine support or perfusion, to improve its longevity to allow the establishment of germinal centers, which generally takes around 4–5 days. Secondary lymphoid organoids, derived from human tonsil, have been described that develop functional germinal centers, but the complex structure of the LN including the supporting stromal cells is probably not fully recapitulated, and these organoids do not have afferent lymphatics or migratory DCs (56). Mechanisms of action of adjuvants on innate cells may be more suited to study in whole LN slices.

Another important limitation of the model is the removal of the blood and lymph circulation. Peripheral immune cells can no longer enter the LN via the afferent lymphatics or high endothelial venules. In a vaccination setting, antigens and adjuvants can be transported by APCs, including DCs, monocytes, and neutrophils (8, 57, 58), from the site of administration to the draining LN, and migrating monocytes and DCs contribute to the cytokine milieu in vivo (30). Our model is suited to studying vaccines and adjuvants that can flow freely to the LNs. We also did not take into account soluble plasma-derived mediators in this project.

The value of this human LN model is in testing the mechanism of action of vaccines and adjuvants in a human setting. This preclinical model holds the potential for comparisons of immunogenicity between different adjuvants and modifications of existing adjuvants by medicinal chemistry, as well as the comparison of modes of action of different vaccine technologies, such as adjuvanted, live attenuated, and mRNA-based vaccines. It provides a benchmark for comparison with other models such as mice, human LN aspirates, or complex blood/lymphoid-derived in vitro models.

## Methods

### Sex as a biological variable.

Our study exclusively examined female human LNs, as males rarely undergo axillary sentinel node biopsies for breast cancer or other causes. Axillary LNs are the draining LNs for vaccines delivered in the upper arm. In the pivotal recombinant zoster vaccine trials, there were no sex-specific effects found at any age including in individuals above 70 YOA (45), therefore, we expect our findings to be relevant to both sexes.

### Human LN explant model.

Human axillary LNs were obtained from patients with clinically node-negative breast cancer who were undergoing sentinel node biopsies and consented to the removal of an additional LN for this study. Additionally, participants had no relevant comorbidities and were not on immunomodulating drugs such as steroids or cytotoxic drugs that could be lymphocytic. The donors ranged from 30–96 YOA, and the LN size ranged from 3–20 mm in the longest dimension. Data were excluded if LN samples had poor viability and when an insufficient number of myeloid cells were recovered from a sample. This most often occurred in LNs that were excessively damaged by cauterization during excision. In addition, data from any participants whose LNs were confirmed pathologically to have cancer were excluded; there were no cases of this in the present study.

Within 60 minutes after surgery, LNs were collected and trimmed of excess fat under a stereo microscope and cut longitudinally into two or three 2 mm thick by 5–7 mm wide slices. These were cultured in 48-well plates, cut face down, on gelfoam (Pfizer) presoaked with DC culture medium (DCM) formulated to support DC viability RPMI (Lonza) supplemented with 10 μM HEPES, 1 mM sodium pyruvate, 1× nonessential amino acids, 0.05 mM gentamicin (all from Gibco, Thermo Fisher Scientific), 50 μM 2-mercaptoethanol, and 10% human serum (both from MilliporeSigma/Merck, v/v), with or without adjuvant. In some instances, a 6 mm cloning cylinder was sealed to the capsule of the LN slice using surgical glue, and the stimulus was applied through this to simulate exposure via the afferent lymphatics. LN slices were cultured for up to 24 hours as indicated in the individual experiments. Supernatants were collected, and cells were either mechanically dissociated for flow cytometric analysis, or tissue slices were fixed or frozen for microscopy. Alternatively to the in situ exposure model, cells were dissociated from fresh LN tissue and immediately assessed by flow cytometry or stimulated in vitro at 1 × 10^6^ cells/mL with adjuvants for 24 hours. The adjuvants used for stimulation were 25 μg/mL AS01, one-quarter of the AS01 concentration administered intramuscularly in humans (AS01_B_), 25 μg/mL MPL formulated in liposomes and 25 μg/mL QS-21 formulated in liposomes (GSK), 25 μg/mL unformulated MPL, 10 μg/mL R848, and 1 μg/mL Pam2Cys (InvivoGen). Unformulated MPL was used throughout. MPL in liposomes was only used in direct comparison with unformulated MPL (Supplemental Figures 7 and 8). DiO or DiD-labeled liposomes (10 mM) (1,2-dioleoyl-sn-glycero-3-phosphocholine [DOPC]/cholesterol 54:45 mol/mol, 1:200 dye/lipid; mean diameter, 200 nm) were provided by Harry Al-Wassiti (Monash University, Melbourne, Victoria, Australia). For in vitro intracellular cytokine staining (ICS) assays by flow cytometry, cells were stimulated for a total of 24 hours at 10 × 10^6^ cells/mL with AS01, R848, or 50 ng/mL PMA/1 μg/mL ionomycin (MilliporeSigma/Merck). BFA (2.5 μg/mL) (MilliporeSigma/Merck) was added after 2 hours or 8–12 hours of culturing.

### Flow cytometry.

Cells were stained with Live/Dead Fixable Viability Stain 700 (BD) for 30 minutes at 4°C. A panel of surface antibodies was then used to stain cells (2.5 × 10^6^ cells/100 μL test), according to standard procedures, in FACSwash (PBS/1% FCS [MilliporeSigma/Merck] with 5 mM EDTA). Cells were fixed with BD Cytofix prior to acquisition. If intracellular staining was required, cells were permeabilized with BD Cytofix/Cytoperm and stained with antibodies prior to acquisition. For intracellular cytokine staining assays including BFA, all antibody staining was conducted intracellularly. Cells were acquired on the BD Symphony flow cytometer, and data were analyzed by FlowJo, version 10.8.1, and GraphPad Prism 9 (GraphPad Software). The following antibodies used were obtained from BD: CD11c BUV661 (B-ly6), CD14 BUV737 (M5e2), CD1a BV510 (HI149), CD1c BV650 (F10/21A3), CD3 BUV496 (UCHT1), CD45 BUV805 (HI30), CD56 BUV563 (NCAM16.2), CD69 BV480 (FN50), CD8 FITC (5C3), CD80 FITC (L307.4), CD83 PE (HB15e), CD86 BV786 (2331 (FUN-1)), HLA-DR BUV395 (G46-6), and IFN-γ PE-Cy7 (B27). The following antibodies from BioLegend were used: CD11b BV711 (ICRF44), CD123 PE-Cy5 (6H6), CD16 BV570 (3G8), CD19 BV750 (HIB19), CD68 APC-Cy7 (Y1/82A), and XCR1 BV421 (ZET). The following antibodies used were from Thermo Fisher Scientific: CD13 PerCP ef710 (WM15), CD169 PE-ef610 (7-239), IL-1β PE (CRM56), IL-12/23p40 ef660 (HP40), and IL-12/IL-23p40 PE (C8.6). Langerin PE-Vio770 (MB22-9F5) antibodies were obtained from Miltenyi Biotec.

### Microscopy imaging.

LN slices stimulated in situ with DiD-labeled liposomes were frozen in OCT. Sections (7 μm thick) were fixed with 2% paraformaldehyde (PFA) and then blocked and permeabilized with PBS, 0.1% saponin, 1% BSA, 10% normal donkey serum, and 1% HEPES. Tissue was incubated with the primary antibodies CD11c (clone 3.9, Invitrogen, Thermo Fisher Scientific) and CD169 (SP216, Merck) for 1 hour at 37°C and with the secondary antibodies donkey anti–mouse Alexa Fluor 555 and donkey anti–rabbit Alexa Fluor 647 (Thermo Fisher Scientific) for 30 minutes at room temperature (RT). The tissue sections were counterstained with DAPI and mounted with ProLong Diamond (Thermo Fisher Scientific). Images were acquired on an Olympus VS-120 Virtual Slide Microscope at ×20 and analyzed using Fiji software.

For imaging mass cytometry (IMC), 5 μm formalin-fixed, paraffin-embedded (FFPE) tissue sections were dewaxed and rehydrated in xylol 3 times for 5 minutes each, in 100% ethanol 3 times for 5 minutes each, in 70% ethanol for 5 minutes, and then PBS. Antigen retrieval was performed in Dako AR Buffer, pH 9.0, at 95°C for 20 minutes in a Biocare Decloaking Chamber NxGen. Slides were blocked with Bloxall (Vector Laboratories) at RT for 10 minutes and then incubated with a metal-conjugated antibody cocktail in TBS-Tris and 1% BSA overnight at 4°C. Slides were washed twice in PBS and 0.1% Triton-X for 8 minutes each. This was repeated with PBS. Nuclei were stained with a DNA intercalator for 30 minutes at RT. Images were acquired on a Hyperion Imaging Mass Cytometer (Standard Biotools).

### Cytokine immunoassays.

The LEGENDplex bead–based multianalyte flow assay kit (Human Inflammation Panel 1, BioLegend) was used to detect a panel of 13 human inflammatory cytokines in culture supernatants as per the manufacturer’s protocol. IL-6 and IL-8 were measured by ELISA (both from BioLegend), as their concentrations exceeded the range of the LEGENDplex assay. For LEGENDplex experiments, plates were acquired on the BD FACSCanto II, and data were analyzed with LEGENDplex Data Analysis software (BioLegend). For all ELISA assays, absorbance was measured on the SpectraMax iD5 Plate Reader, and data were analyzed using GraphPad Prism 9.20 (GraphPad Software).

### T cell alloproliferation assay.

Following mock or AS01 treatment of LN slices for 20 hours, cells were isolated from the tissue by digestion with 3 mg/mL collagenase type IV (Worthington) with 250 U/mL DNAse (Roche) for 40 minutes at 37°C. Cells were washed and stained with the viability dye FVS700 (BD) and CD3 APC-Vio770, CD19 APC-Vio770, HLA-DR PerCP (all Miltenyi Biotech), CD14 BV480, CD11c BB515, CD1c BV650, CD83 PE (all from BD), XCR1 APC, CD123 PE-Cy5 (all from BioLegend), and CD169 PE-efluor610 (Thermo Fisher Scientific) antibodies. DCs were then sorted on a BD Influx cell sorter (BD Biosciences) by gating on live CD3^–^, CD19^–^, CD14^–^, autofluorescence^–^, HLA-DR^+^ cells. DCs (10,000–15,000 cells) were cocultured at a ratio of 1:2.5 with CellTrace Violet–labeled (Thermo Fisher Scientific) heterologous naive CD4^+^ T cells (combined from three donors, previously isolated with Miltenyi Naive CD4 T cell Kit) in DCM for 5.5 days. As a positive control, T cells were cultured with anti-CD3 and anti-CD28 monoclonal antibodies at 1 and 5 μg/mL, respectively, or in media alone as a negative control. On day 6, T cells were analyzed on a BD Fortessa flow cytometer for proliferation, measured by CellTrace Violet dilution.

### Statistics.

Statistical analyses were performed using GraphPad Prism 9.0 and R Studio, version 4. Data were assumed not to follow a normal distribution on the basis of visual inspection or failure of normality tests. Therefore, nonparametric tests were used throughout or, in some cases, data were log_e_ transformed to approximate normality, and equivalent parametric tests were applied with Bonferroni-Dunn corrections for multiple comparisons, as described in the figure legends. A GEE model was used to model the immune response as measured by cytokine levels. The model was equipped with a log-normal link function, an exchangeable correlation structure of the multiple treatments received by each donor, and adjustment for the fixed effect of age. When multiple hypotheses were adjusted for, the Bonferroni-Dunn method was used. A *P* value of less than 0.05 represented statistical significance.

### Study approval.

This study was approved by the Western Sydney Local Health District (WSLHD) Human Research and Ethics Committee (2019/ETH01894, 2021/ETH12256), and informed, written consent was obtained from all participants prior to the collection of tissue.

### Data availability.

All data are available in the main text, supplemental materials, and the Supporting Data Values file.

## Author contributions

ALC, KJS, AMD, MC, and STT conceptualized the study. KJS, VVS, ALC, KMB, VAV, HB, JE, MC, EE, JF, and FM designed the study methodology. VVS, KJS, VAV, EBD, DJT, and JE performed experiments and data visualization. ALC, KJS, and MC acquired funding and were responsible for project administration. KJS and ALC supervised the study. KJS, VVS, and ALC wrote the original draft of the manuscript. KJS, VVS, ALC, MC, ST, and AMD reviewed and edited the manuscript.

## Supplementary Material

Supplemental data

Supporting data values

## Figures and Tables

**Table 1:**
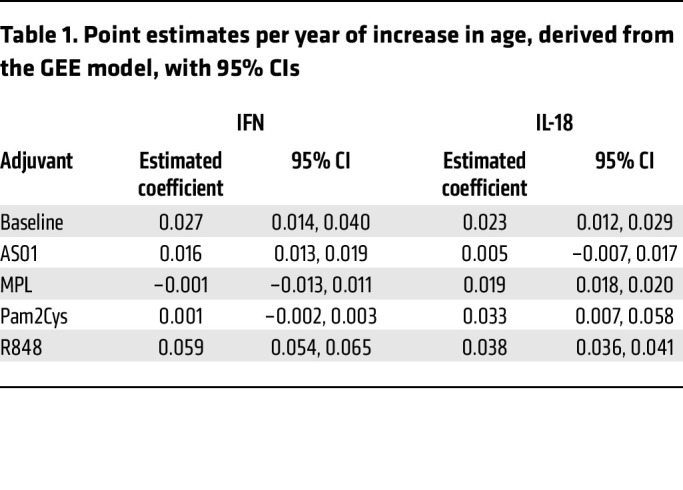
Point estimates per year of increase in age, derived from the GEE model, with 95% CIs

## Version 1 09/24/2024

In-Press Preview

## Version 2 11/15/2024

Electronic publication
