# Supplementary material for: Innate immune cell activation by adjuvant AS01 in human lymph node explants is age independent
Source: J Clin Invest. 2024 Sep 24;134(22):e174144. doi: 10.1172/JCI174144 (PMC11563676; doi:10.1172/JCI174144)
Supplement: Supplemental data [file jci-134-174144-s259.pdf]

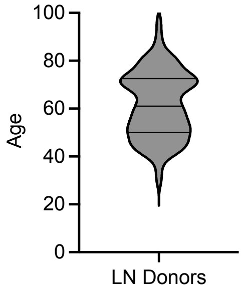

Figure S1: Age distribution of lymph node donors. Violin plot showing median and interquartile range of LN donor ages.

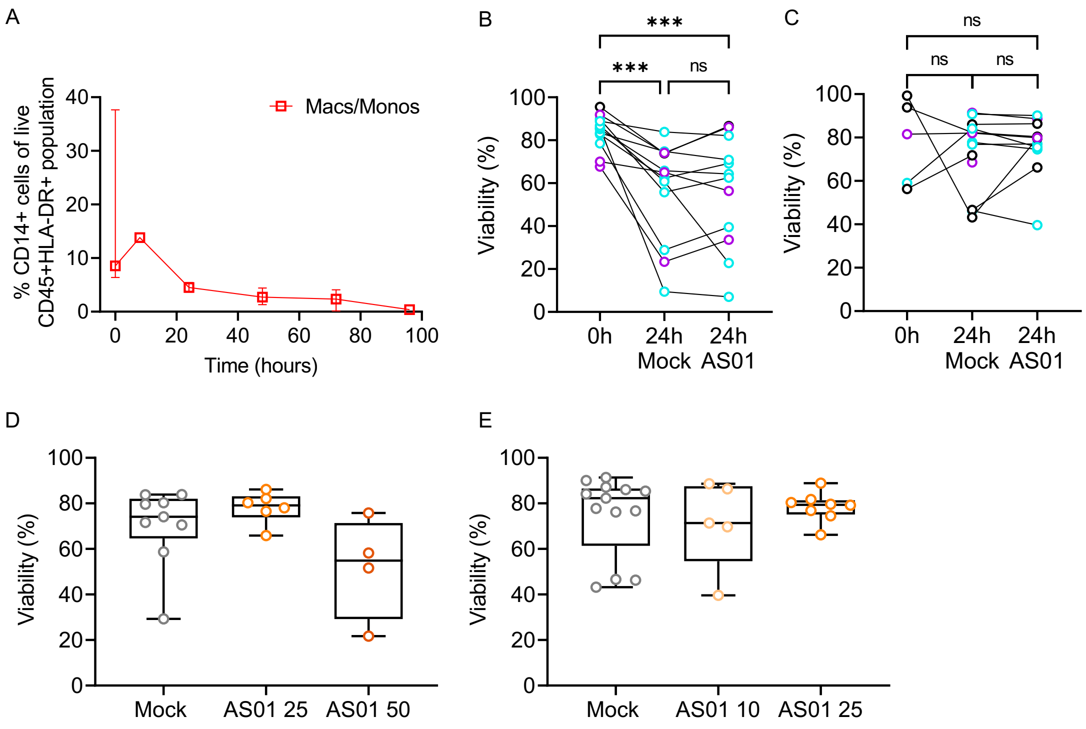

Figure S2: Viability of lymph node immune cells in the *in situ* explant model vs *in vitro* cultured cells. Slices of human LNs were mock treated or bathed in AS01 for 24 h. Cells were mechanically dissociated from the tissue and immune cell viability was determined by flow cytometry. Alternatively dissociated LN cells were exposed to AS01 *in vitro*. **(A)** Frequency of CD14+ monocytes/macrophages of the live CD45+HLA-DR+ population over time in LN slices cultured without stimuli (n=2-3). Median with interquartile range plotted. **(B)** Viability of CD45+ immune cells from LN slices that were AS01 or mock treated *in situ* for 24 h, compared to viability of fresh LN cells (0 h). **(C)** Viability of isolated LN CD45+ immune cells that were AS01 or mock treated *in vitro* for 24 h, compared to fresh LN cells (0 h). Repeated measures ANOVA with Tukey's multiple comparisons test was performed. *** $p < 0.001$, ns = non-significant. Donor age: purple ≤50 years; black 51-65 years; aqua >65 years. Toxicity study of AS01 **(D)** in LN slices and **(E)** in isolated cells up to 50 µg/mL. Viability of CD45+ immune cells is shown, where AS01 was tolerated up to 25µg/mL. Median and interquartile range are shown. All comparisons ns by Wilcoxon matched pairs signed rank test.

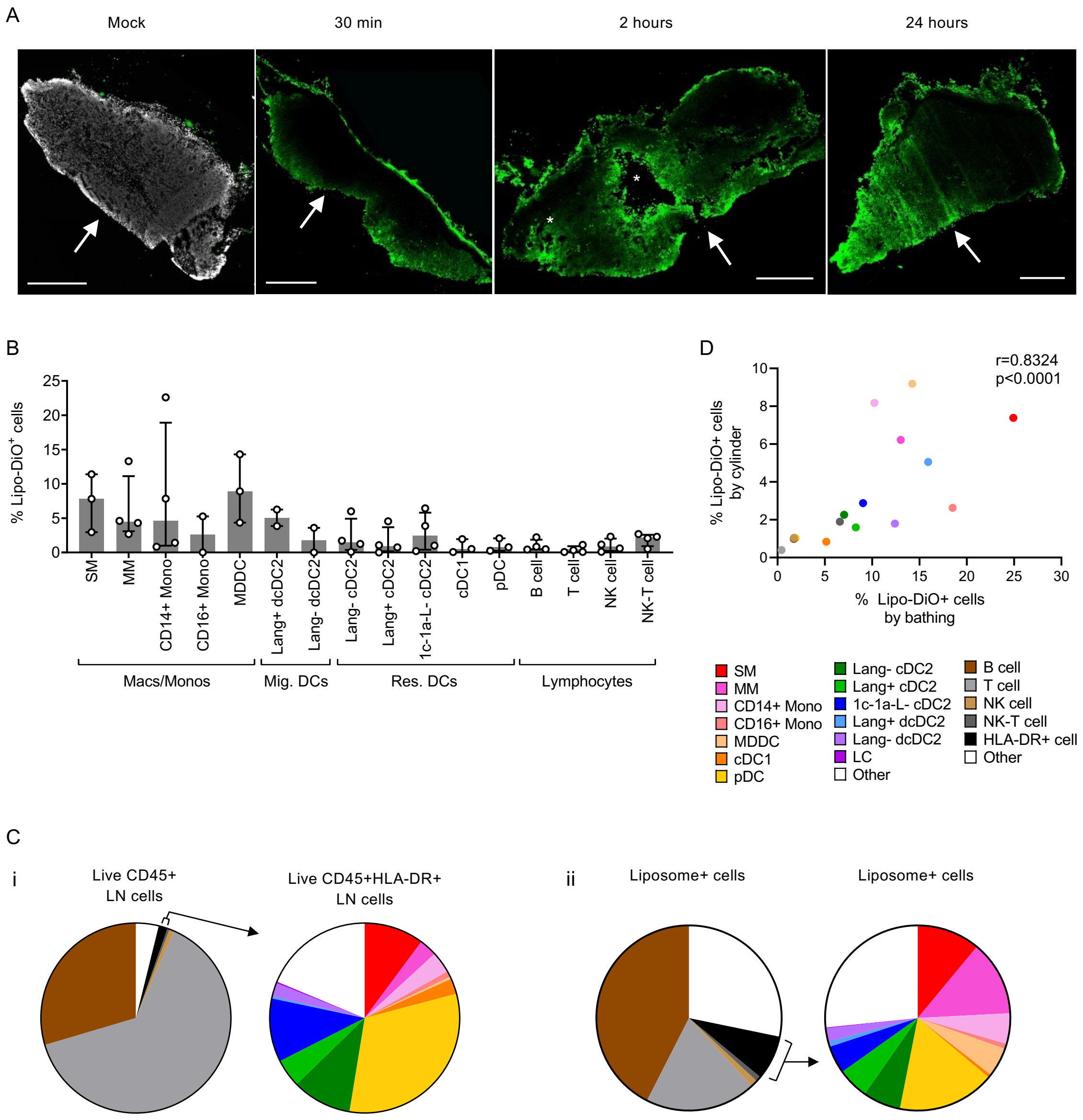

Figure S3: Fluorescent liposomes, a model for AS01, are preferentially taken up by subcapsular sinus lining macrophages following cylinder and bathing exposure *in situ*. Slices of human LNs were exposed to DiO-labeled liposomes for 2 h via the bathing (A and D) or cylinder application (B-D) methods. The 30 min timepoint from the time series in (A) is also shown in Figure 2B. **(A)** Fluorescence microscopy showing liposome (green) penetration of LN slice over time. Bathed face is indicated by white arrow. Cell nuclei are shown in the mock (DAPI, grey). Asterisks indicate fat deposits within the LN which likely facilitated liposome uptake. Scale bar represents 500 µm. **(B)** Comparison of liposome uptake (% positive cells) between LN cell subsets after 2 h cylinder exposure, measured by flow cytometry (n = 2-4). Median + interquartile range is shown for cell subsets of the major groups: macrophages/monocytes (macs/monos), migratory dendritic cells (mig. DCs), resident dendritic cells (res. DCs) and lymphocytes. Mixed effects analysis with Tukey's multiple comparisons test was performed. **(C)** Proportion analysis of immune cell subsets **(i)** present in the LN (n = 50) and **(ii)** making up the total liposome+ fraction after 2 h exposure (n = 5), showing cell subsets as a percentage of total live, CD45+ immune cells and myeloid cell subsets as a percentage of HLA-DR+ cells. **(D)** Spearman's correlation of liposome uptake by various cell subsets via cylinder and bathing exposure.

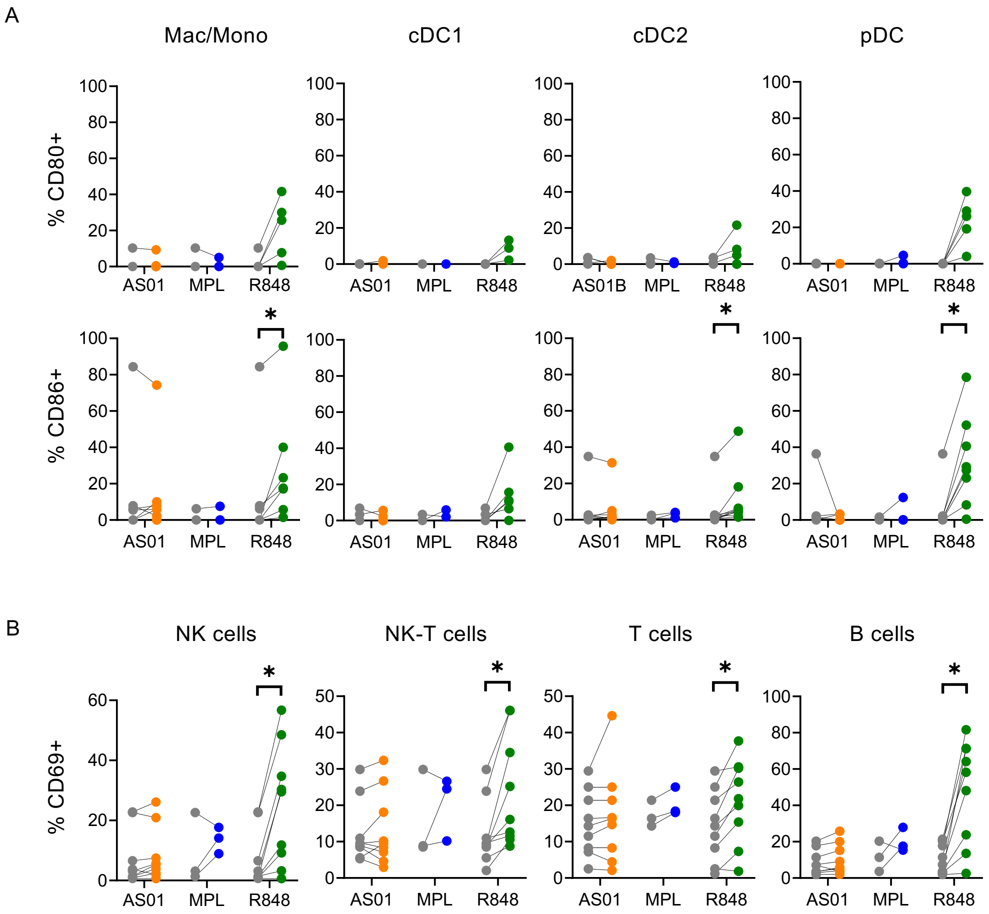

Figure S4: AS01 does not induce maturation of macrophages and dendritic cells or activation of lymphocytes *in vitro* in dissociated human LN cells. Cells from whole human LNs were mechanically dissociated and stimulated *in vitro* for 24 h with adjuvants AS01 (orange), MPL (blue) and R848 (green) or mock treated (grey). The percent of **(A)** macrophage and dendritic cell populations expressing maturation markers CD86 (AS01 n = 6-9, MPL n = 2-3, R848 n = 6-9) and CD80 (AS01 n = 3-6, MPL n = 1-3, R848 n = 3-6), and **(B)** NK cells and lymphocytes expressing the early activation marker CD69 (AS01 n = 9, MPL n = 3, R848 n = 9) were assessed by flow cytometry. Wilcoxon matched-pairs signed rank tests were applied with Bonferroni-Dunn correction for multiple comparisons. * $p < 0.05$.

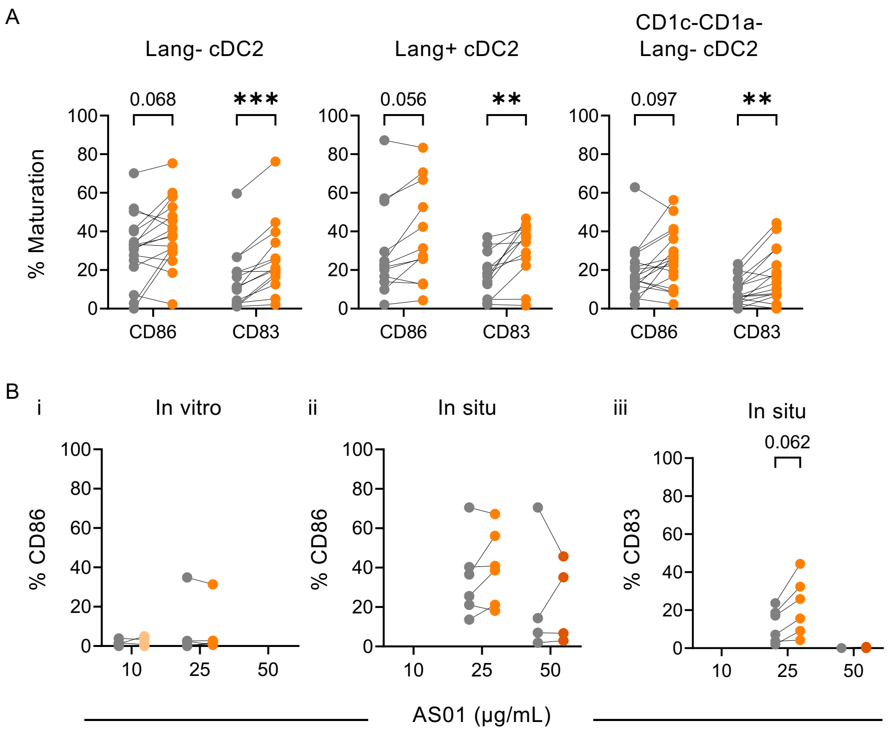

Figure S5: AS01 induces maturation of subsets of resident type II conventional dendritic cells, only *in situ* at the optimal concentration. Slices of human LNs were bathed in adjuvants AS01 (orange) or mock treated (grey). Cells were then mechanically dissociated from the LN tissue and the percent of different dendritic cell populations expressing maturation markers CD83 and CD86 was assessed via flow cytometry. **(A)** Maturation in cDC2 subsets after *in situ* exposure to 25 µg/mL AS01 (n = 8-17). Analyses by Wilcoxon matched-pairs signed rank tests were applied with Bonferroni-Dunn correction for multiple comparisons. ** p < 0.01, *** p < 0.001. **(B)** Titration of AS01 showing expression of **(i)** CD86 *in vitro* (10 µg/mL n = 4; 25 µg/mL n = 6); **(ii)** CD86 *in situ* (25 µg/mL n = 6; 50 µg/mL n = 4); **(iii)** CD83 *in situ* (25 µg/mL n = 6; 50 µg/mL n = 3) on all CD11c+ cDC2s.

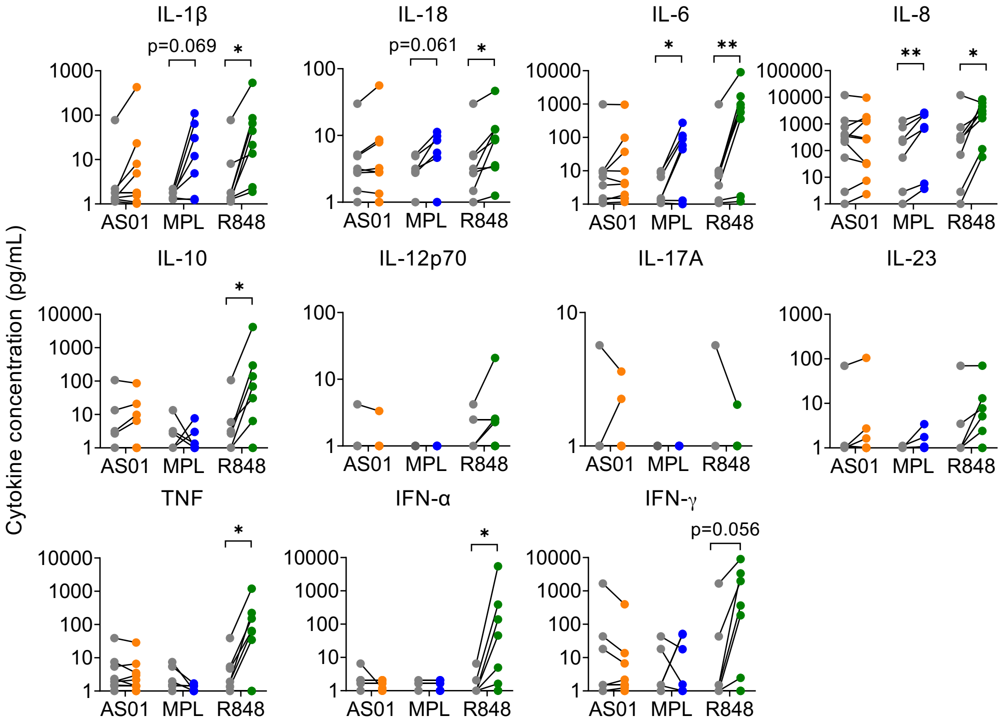

Figure S6: AS01 does not induce the production of key pro-inflammatory cytokines from total lymph node immune cells *in vitro*. Cells were mechanically dissociated from whole human LNs and exposed to adjuvants AS01 (orange, n = 10), MPL (blue, n = 7) and R848 (green, n = 8) in culture for 24 h. Cytokine concentrations in culture supernatants were determined by LEGENDplex and compared to their donor matched mock (grey) samples. Data were $\log_e$ transformed to approximate normality then paired t-tests corrected for multiple comparisons using the Bonferroni-Dunn method were applied. * $p < 0.05$, ** $p < 0.01$.

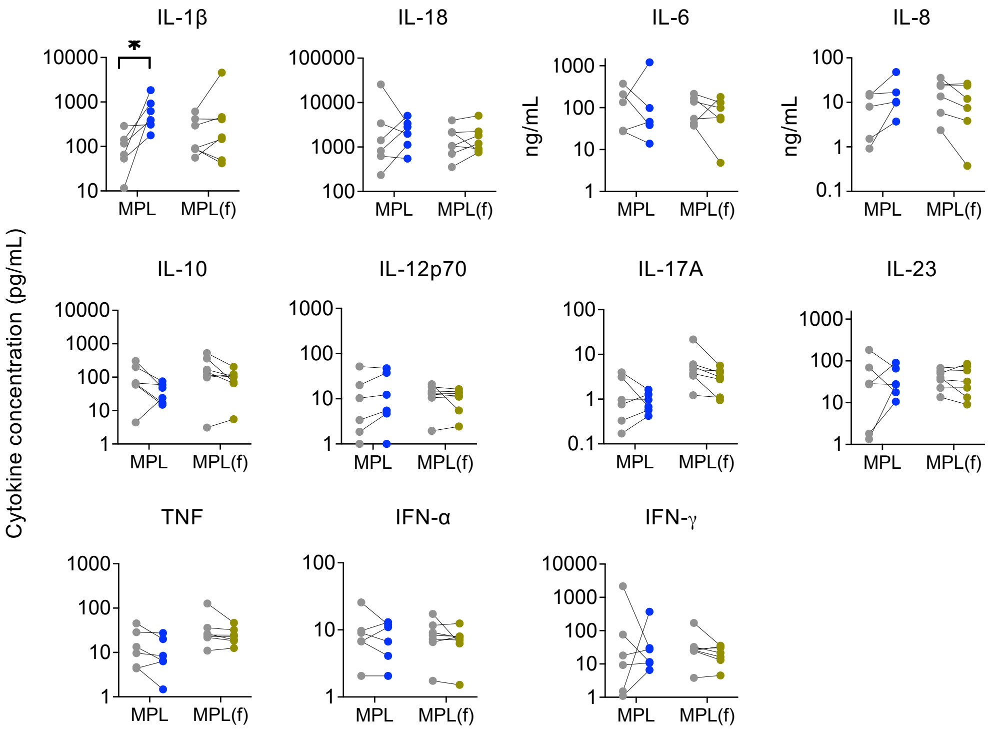

Figure S7: Comparison of cytokine induction by unformulated MPL and MPL formulated in liposomes. Slices of human LNs were bathed in unformulated MPL (blue, n = 6) or MPL formulated (f) in liposomes (olive, n = 7) for 24 h. Cytokine concentrations in culture supernatants were determined by LEGENDplex and compared to their donor matched mock (grey) samples. IL-6 and IL-8 was measured by ELISA. Data were $\log_e$ transformed to approximate normality and a repeated measures ANOVA showed no significant interaction between the type of MPL and the effect of MPL on any of the cytokines. A t-test was performed on transformed data to compare mock vs. treatment for each type of MPL. *p<0.05

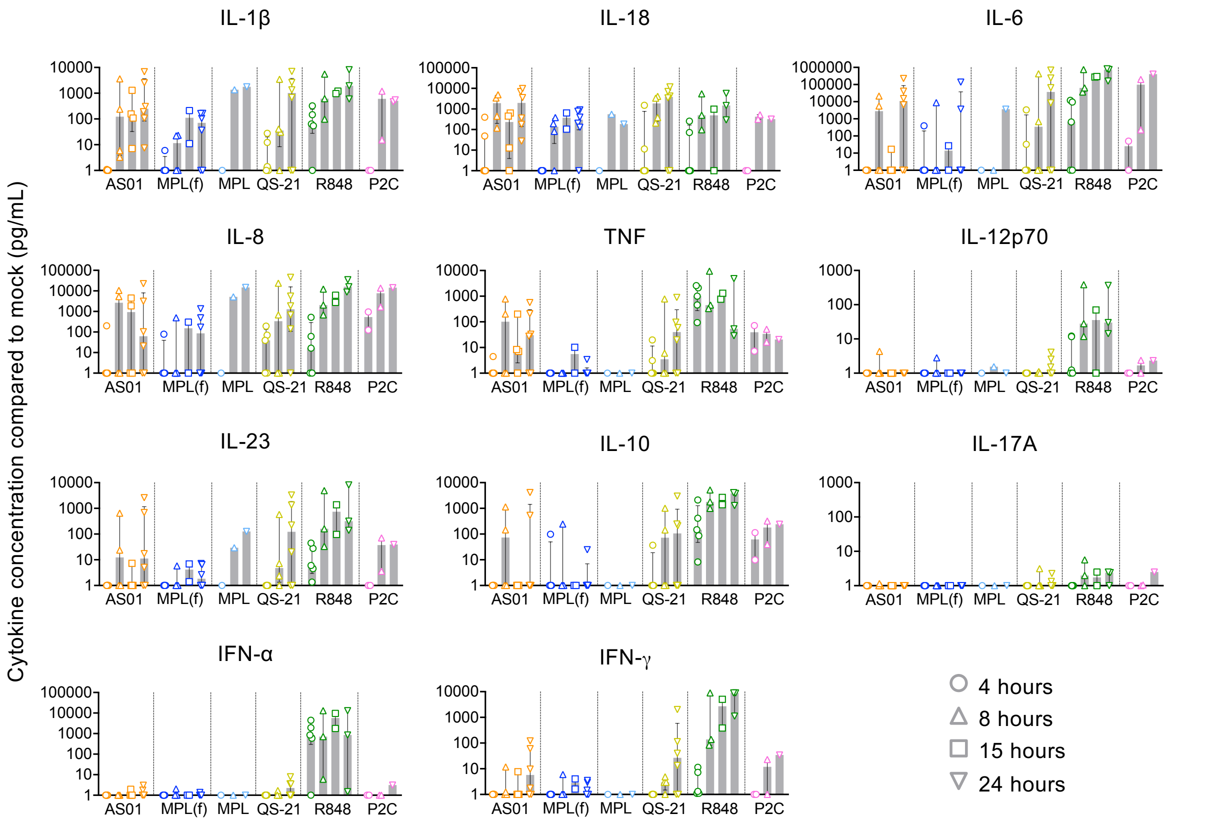

Figure S8. Timecourse of LN cytokine response to adjuvants. Slices of human LNs were bathed in AS01, MPL formulated in liposomes (MPL(f)), unformulated MPL (MPL), QS-21, R848 or Pam2Cys (P2C). Supernatants were sampled over a 24 h period but not all LNs could be sampled at every timepoint. Cytokine concentrations in culture supernatants were determined by LEGENDplex and IL-6 and IL-8 were measured by ELISA. Donor matched mock values were subtracted and values <1 were arbitrarily assigned to 1 pg/mL. Medians and interquartile range are shown.

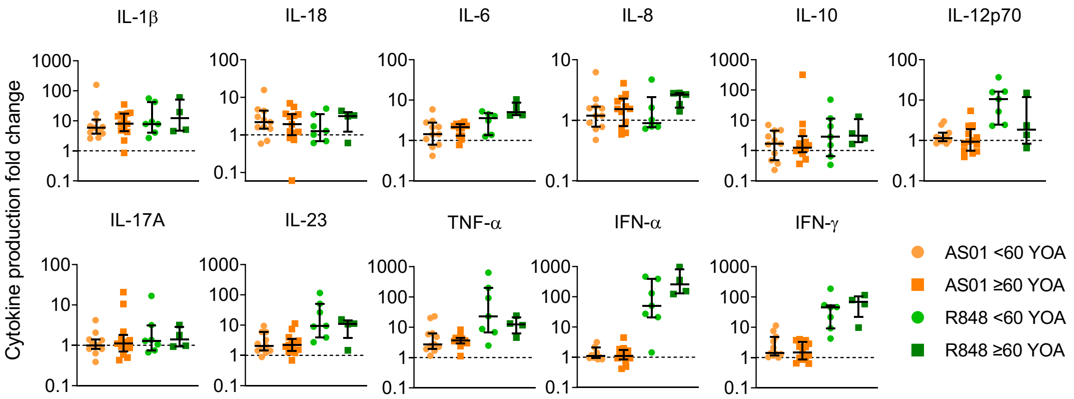

Figure S9: Fold change in cytokine levels in responses to AS01 and R848 in young (< 60 YOA) and old (> 60 YOA) LN donors. Slices of human LNs were stimulated *in situ* for 24 h with AS01 (orange) and R848 (green) and comparisons made between young (<60 YOA, circles) and old (≥60 YOA, squares) donors. Fold change in cytokine production compared to donor-matched mock samples is plotted. (AS01; young n = 10-12, old n = 9-10. R848; young n = 5-6, old n = 3-4). Mann Whitney tests corrected for multiple comparisons using the Bonferroni-Dunn method were applied. Medians with interquartile range are indicated throughout.

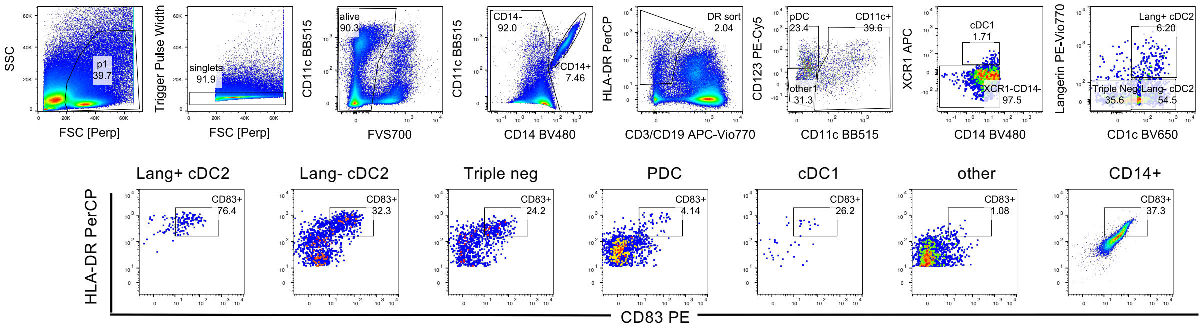

Figure S10: FACS sort strategy and phenotype of DCs used in T cell proliferation assays. DCs were FACS sorted from dissociated LN cells from AS01 or mock treated LN slices. Live, CD14-, CD3-, CD19-, HLA-DR+ cells were collected and used in T cell proliferation assays. The DC subsets identifiable within this population and their expression of CD83 is shown for a representative AS01-treated donor.
